# Effect of Waste Calcareous Opoka Rock on the Rheological, Physical, and Durability Properties of Thin-Layer Renders for External Thermal Insulation Composite Systems

**DOI:** 10.3390/ma19183903

**Published:** 2026-09-14

**Authors:** Danuta Barnat-Hunek, Małgorzata Grzegorczyk-Frańczak, Nikolas Robaczyński, Wiktoria Puch

**Affiliations:** Faculty of Civil Engineering and Architecture, Lublin University of Technology, ul. Nadbystrzycka 40, 20-618 Lublin, Poland; m.grzegorczyk@pollub.pl (M.G.-F.); s98183@pollub.edu.pl (N.R.); s98180@pollub.edu.pl (W.P.)

**Keywords:** calcareous opoka rock, thin-layer renders, durability, microstructure, adhesion

## Abstract

**Highlights:**

- Waste calcareous opoka rock (WCOR) was assessed as a functional mineral component of thin-layer ETICS plasters.
- WCOR markedly improved the freeze–thaw and salt-crystallisation resistance of renders.
- SEM/EDS revealed matrix-dependent differences in local morphology and aggregate–binder interfacial contact.
- Multi-criteria analysis distinguished between durability-driven and balanced WCOR optima.

**Abstract:**

Waste calcareous opoka rock (WCOR) was investigated as a mass-based replacement for 0.5–1.5 mm granulated dolomite aggregate in thin-layer silicate and mineral renders intended for external thermal insulation composite systems (ETICS). Eight formulations with replacement levels ranging from 0% to 100% were evaluated in terms of fresh-state properties, moisture transport, adhesion, freeze–thaw and sodium sulphate crystallisation resistance, and microstructure using SEM/EDS. Increasing WCOR content reduced fresh-mixture density and flow diameter while increasing resistance to manual surface texturing. In silicate renders, capillary water absorption decreased by 84%, from 1.55 kg/(m^2^·24 h) for the WCOR-free reference to 0.25 kg/(m^2^·24 h) at 75% replacement. Conversely, in mineral renders, it increased by 52%, from 17.2 to 26.2 kg/(m^2^·24 h) at complete replacement. At 100% replacement, freeze–thaw resistance increased from 150 to 308 cycles in silicate renders and from 105 to 251 cycles in mineral renders, while salt-crystallisation resistance increased from 25 to 52 and from 16 to 52 cycles, respectively. Adhesion increased from 0.27 to 0.30 N/mm^2^ in silicate renders but decreased from 0.31 to 0.19 N/mm^2^ in mineral renders. SEM/EDS showed matrix-dependent differences in local morphology and aggregate–binder interfaces. Multi-criteria assessment identified 75% replacement as the most balanced option for silicate renders, whereas 25–50% provided the preferred compromise for mineral renders. Overall, the influence and preferred content of WCOR were strongly matrix-dependent.

## 1. Introduction

Limestone is a sedimentary rock composed predominantly of calcium carbonate, although its engineering properties are also influenced by marly and clayey constituents, porosity, cohesion, and structural heterogeneity [1]. The extraction and processing of carbonate rocks generate mineral waste, including fine fractions, dusts, slurries, and rock fragments. Owing to their limited opportunities for direct reuse and the difficulties associated with transport and storage, these materials are frequently disposed of in landfills [1,2,3]. Their use as aggregate or mineral additions in cement-based materials may reduce both the consumption of natural resources and the amount of waste requiring disposal.

Limestone cutting waste, stone dust, and carbonate slurries have been investigated as partial replacements for natural sand and fine aggregate in concrete and mortar. Depending on their particle-size distribution, mineralogical composition, morphology, and replacement level, these materials may contribute to particle packing and act as mineral fillers or nucleation sites for hydration products [2,4]. Their influence on composite properties, however, depends strongly on the porosity, water absorption, particle geometry, and mineralogical composition of the waste.

One potential secondary mineral resource is calcareous opoka, a porous carbonate–siliceous rock consisting of a carbonate matrix with siliceous, clayey, and detrital constituents [5]. Opoka is a highly fossiliferous marine spiculitic rock characteristic of Upper Cretaceous deposits occurring in Poland and other parts of Europe. The material investigated in this study originated from Kazimierz Dolny, a town located in the Lublin Voivodeship of eastern Poland, within the Middle Vistula River valley. This area contains well-developed outcrops of Upper Maastrichtian opoka, which has been used as a local building material since the Middle Ages [6].

Opoka from the Kazimierz Dolny region is generally characterised by a bulk density of approximately 1.4–1.6 g/cm^3^, porosity reaching approximately 40%, and compressive strength ranging from approximately 10 to 30 MPa [7,8]. Its porous and heterogeneous structure affects its water absorption, strength, and behaviour under moisture exposure. Clay minerals, particularly phases containing smectitic layers, may adsorb water and undergo swelling, which can weaken internal bonds in the intact rock [9,10,11,12,13]. Repeated wetting–drying, freeze–thaw, and salt-crystallisation exposure may further contribute to its deterioration [6,7,14,15]. However, the behaviour of crushed opoka particles embedded in a binder matrix cannot be inferred directly from the behaviour of the intact rock.

Waste calcareous opoka rock (WCOR) may be used as a substitute for conventional mineral aggregate in mortars and renders. Such use could reduce the consumption of natural dolomite aggregate and support the management of locally generated construction waste. At the same time, the high water absorption, irregular geometry, and developed surface of WCOR particles may affect the water demand, density, consistency, and application behaviour of fresh render mixtures. In hardened renders, WCOR may also influence liquid-water uptake, water-vapour transport, adhesion to the substrate, and resistance to cyclic environmental exposure.

Thin-layer renders constitute the outer protective and decorative layer of external thermal insulation composite systems (ETICS) and are directly exposed to rainfall, temperature variations, ultraviolet radiation, freezing, and atmospheric contaminants. Previous investigations of ETICS have focused mainly on commercially available mineral, acrylic, silicate, and silicone renders and their behaviour under moisture exposure, temperature variations, artificial ageing, and environmental degradation [16,17,18,19,20]. Field observations of 378 buildings indicated an average ETICS service life of approximately 21 years, demonstrating the importance of the properties and durability of the external render layer [21].

Despite this research, the use of WCOR as a replacement for conventional aggregate in thin-layer renders remains largely unexplored. In particular, no systematic comparison has been reported of the influence of the same WCOR aggregate on silicate and mineral renders prepared and tested under comparable conditions. It therefore remains unclear how increasing WCOR content simultaneously affects application-related behaviour, moisture transport, adhesion, and resistance to freeze–thaw cycling and salt crystallisation. This combined assessment is important because a replacement level that improves one durability parameter may adversely affect another property and may not provide the most balanced overall performance.

The hypothesis of this study was that replacing dolomite aggregate with WCOR would produce changes in fresh-state, transport, adhesion, and durability properties whose direction and magnitude would depend on the render matrix. Accordingly, the same WCOR replacement level was not expected to provide equivalent performance in silicate and mineral renders. This hypothesis concerns the overall effect of WCOR on render performance and does not presume chemical reactivity of the waste aggregate.

The objective was to determine the effects of partial and complete mass-based replacement of 0.5–1.5 mm granulated dolomite aggregate with WCOR in thin-layer silicate and mineral renders. The experimental programme included fresh-mixture density, consistency, application-related behaviour, capillary water absorption, water-vapour permeability, adhesion, resistance to freeze–thaw cycling and sodium sulphate crystallisation, accelerated ageing, and qualitative SEM/EDS observations. Multi-criteria and Pareto analyses were used as supplementary tools to distinguish formulations maximising cyclic durability from those providing a more balanced combination of durability, moisture transport, adhesion, and application-related properties.

The renders were tested on rigid concrete substrates under controlled laboratory conditions. Therefore, the results characterise the comparative behaviour of render–concrete assemblies and should not be interpreted as a direct assessment of complete ETICS, which also comprise thermal insulation, an adhesive or reinforced base coat, a reinforcement mesh, primer, and a finishing render.

## 2. Materials and Methods

### 2.1. Materials

Omyadol^®^ 40-JA is a finely ground, high-purity white dolomitic mineral filler manufactured by Omya Sp. z o.o. (Jasice/Wojciechowice, Poland). It is not a chemical admixture or polymeric binder, but a powdered mineral constituent composed predominantly of dolomite, i.e., calcium magnesium carbonate, CaMg(CO_3_)_2_. The product is characterised by low binder absorption and is commonly used as a mineral filler and white mineral pigment.

In mortars and renders, Omyadol^®^ 40-JA increases the proportion of fine mineral particles, improves particle-size grading and matrix packing density, and may enhance workability and surface smoothness. It also affects the viscosity, consistency, and stability of the fresh mixture, while contributing to the light colour of the final product.

Omyadol^®^ 0.5–1 and Omyadol^®^ 1.0–1.5 are natural dolomite aggregates with nominal particle-size ranges of 0.5–1.0 mm and 1.0–1.5 mm, respectively. They were used as structural mineral aggregate fractions in the render formulations. The particle density, loose bulk density, and tamped apparent density were determined separately for both Omyadol^®^ fractions. For Omyadol^®^ 0.5–1.0 mm, the particle density was 2.90 g/cm^3^, the loose bulk density was 1.40 g/cm^3^, and the tamped apparent density was 1.70 g/cm^3^. The same values were obtained for Omyadol^®^ 1.0–1.5 mm: 2.90, 1.40, and 1.70 g/cm^3^, respectively. The particle-density values were taken from the manufacturer’s technical data determined in accordance with ISO 787-10 [22], whereas the loose bulk density and tamped apparent density were determined experimentally. Based on our own research, the following data were obtained: a bulk density of 1.40 g/cm^3^ and a tamped apparent density of 1.70 g/cm^3^ for both aggregate fractions. The Omyadol^®^ 0.5–1 aggregate grain size is presented in Table 1 (own research).

Waste calcareous opoka rock (WCOR).

**Figure 1:**
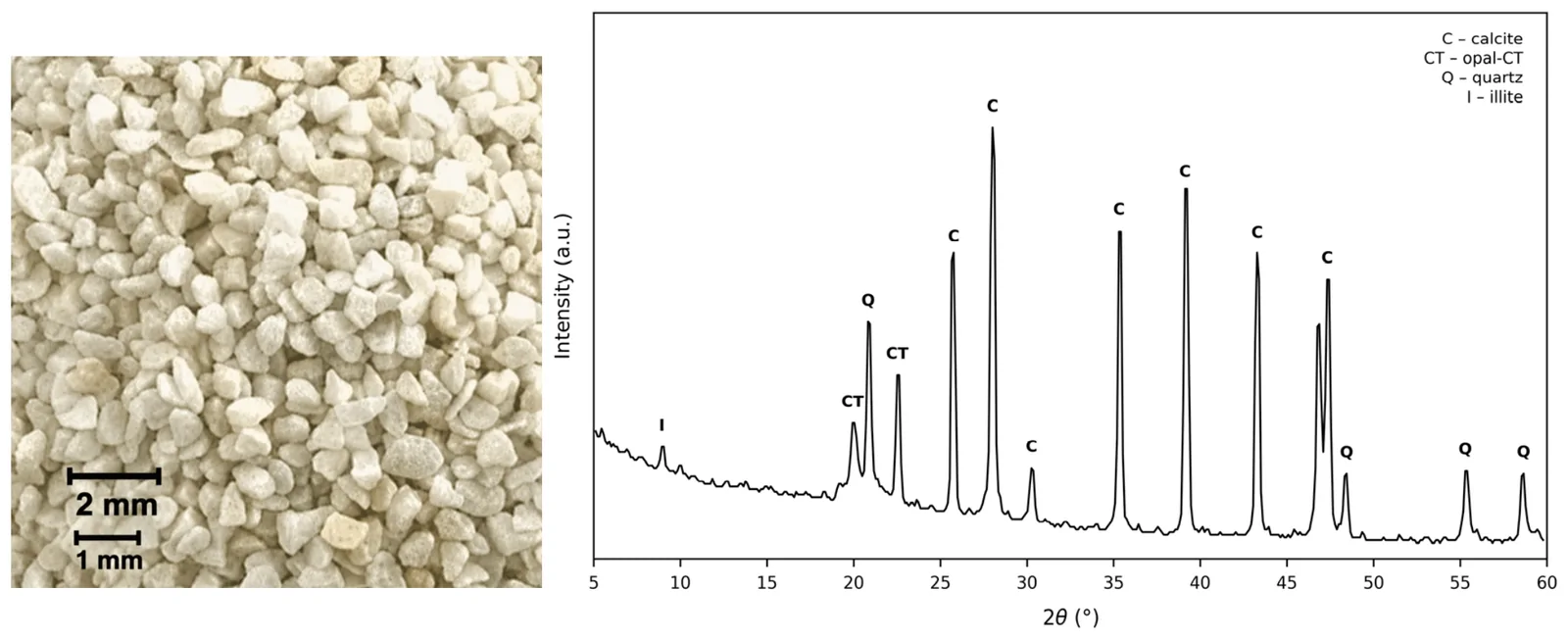
Appearance of waste calcareous rock (1–1.5 mm) and its X-ray diffraction patterns.

**Figure 2:**
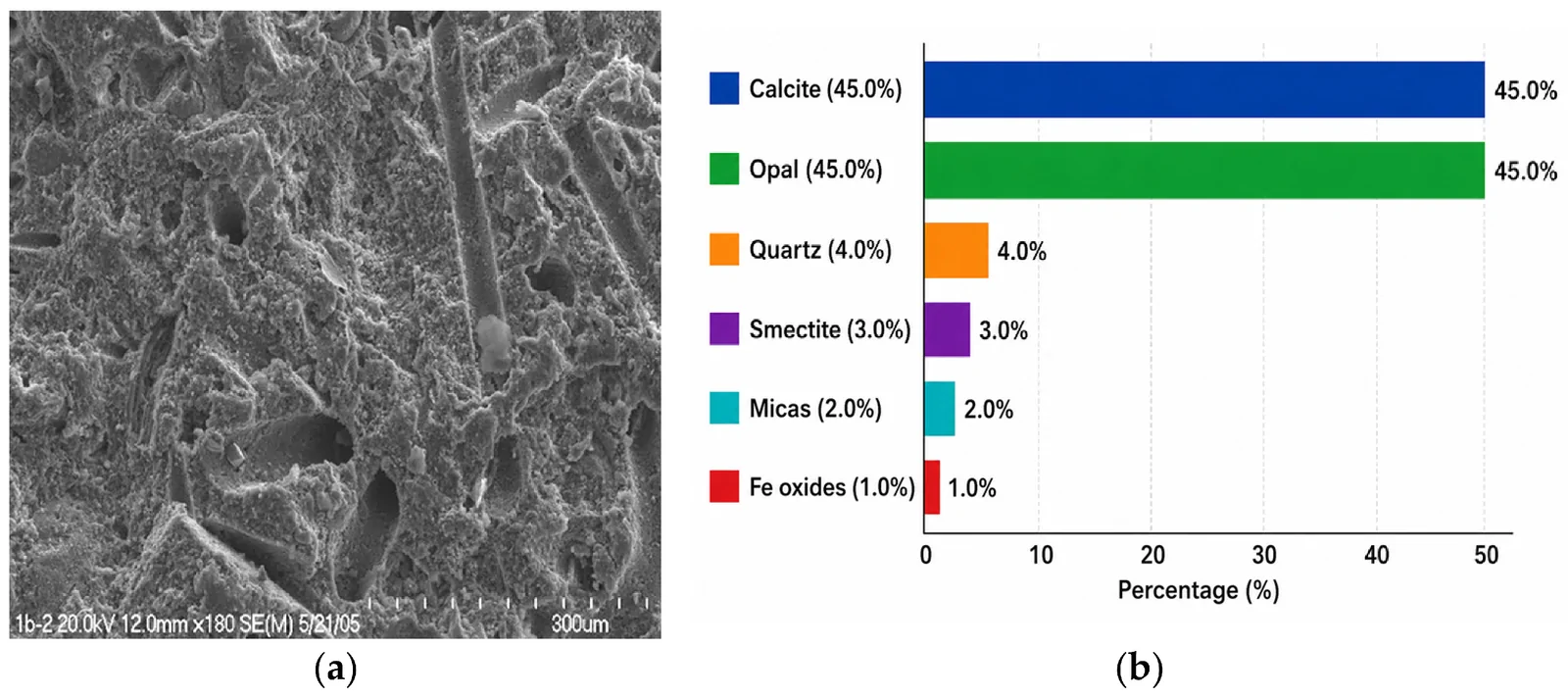
Characterisation of WCOR: (**a**) SEM micrograph of the WCOR microstructure; (**b**) estimated relative contents of the mineral phases identified by XRD analysis.

The calcareous opoka rock used in this study was obtained as construction waste in the form of rubble generated during building works in Kazimierz Dolny, Poland (Figure 1). X-ray diffraction analysis was used to identify the mineral phases present in WCOR. The relative phase contents were estimated semi-quantitatively using the automatic analysis function implemented in X’Pert HighScore software (version 4.1). The results should therefore be interpreted as approximate phase proportions rather than as a full quantitative mineralogical analysis (Figure 2).

Clay minerals were also identified, represented mainly by smectite and illite (Figure 2).

Semi-quantitative mineralogical analysis indicated that calcite and opal were the principal constituents of the investigated opoka, each accounting for 45% of the identified mineral phases. The remaining constituents comprised quartz (4%), smectite (3%), micas (2%), and iron oxides (1%), as shown in Figure 2. The investigated opoka exhibited a non-oriented and heterogeneous bio-organic structure. Fragments of sponge spicules and less frequent foraminiferal remains were embedded in a micritic–siliceous matrix. The presence of smectite may increase the moisture sensitivity of the rock. The crushed WCOR was sieved into two particle-size fractions, 0.5–1.0 mm and 1.0–1.5 mm, corresponding to the nominal particle-size ranges of the Omyadol^®^ aggregates being replaced. The physical properties of the aggregate WCOR were a specific density of 2.73 g/cm^3^, bulk density of 1.57 g/cm^3^, and water absorption rate of 22% [23].

ARBOCEL^®^ B 400 by J. Rettenmaier and Söhne GmbH (JRS) (Rosenberg, Germany) is a high-purity, long cellulose fibre with an average fibre length of approximately 900 µm, used as a rheological stabiliser and micro-reinforcing additive. This material is neither a binder nor a conventional chemical admixture, but rather a functional microfibrous filler and reinforcing agent. Its principal characteristics are as follows: cellulose content of approximately 99.5%; average fibre length of approximately 900 µm; ash content of approximately 0.3%; pH of a 5% aqueous suspension of approximately 5–7; and a physical form consisting of white, elongated cellulose fibres.

Within the formulation, the cellulose fibres can increase the viscosity and stability of the fresh mixture, reduce the sedimentation of mineral fillers, improve water retention, limit sagging on vertical surfaces, mitigate drying-shrinkage cracking, and reinforce the coating structure through the formation of a spatial fibre network. The cellulose fibres form a three-dimensional network within the material, thereby stabilising the fresh formulation and promoting more uniform drying. It should, however, be emphasised that cellulose is hydrophilic. Excessive fibre content may therefore increase water demand, increase viscosity, and affect the water absorption of the hardened material. These effects may be partially compensated for by the presence of the acrylic binder and silicone additive.

A total of eight render formulations were developed, comprising four silicate renders and four mineral renders.

Silicate renders:

S1—Reference formulation developed by a commercial manufacturer, containing 100% granulated dolomite aggregate;

S2—Formulation containing 50% granulated dolomite aggregate and 50% waste calcareous opoka rock (WCOR);

S3—Formulation containing 25% granulated dolomite aggregate and 75% WCOR;

S4—Formulation containing 100% WCOR.

Mineral renders:

M1—Reference formulation developed by a commercial manufacturer, containing 100% granulated dolomite aggregate;

M2—Formulation containing 75% granulated dolomite aggregate and 25% WCOR;

M3—Formulation containing 50% granulated dolomite aggregate and 50% WCOR;

M4—Formulation containing 100% WCOR.

The formulations were selected to investigate the effect of varying amounts of the WCOR additive on the properties of thin-layer plasters in the ETICS. The percentage compositions of the mixtures are shown in Table 2 and Table 3. In mineral renders, the composition was expressed as mass percentages relative to the total mass of the dry mixture. Mixing water was not included in the 100% formulation and was added separately during mortar preparation. Based on preliminary laboratory trials, the water content was set at 22% by mass of the dry mixture.

The compositions of the silicate renders are expressed as mass percentages relative to the total mass of the wet formulation, including water and liquid constituents. The compositions of the mineral renders are expressed as mass percentages relative to the total mass of the dry mixture. Mixing water was not included in the tabulated 100% of the mineral formulations and was added separately at 22% by mass of the dry mixture. All WCOR replacement levels were calculated on a mass basis relative to the combined mass of the replaced Omyadol^®^ 0.5–1 and Omyadol^®^ 1.0–1.5 fractions.

### 2.2. Methods

**Figure 3:**
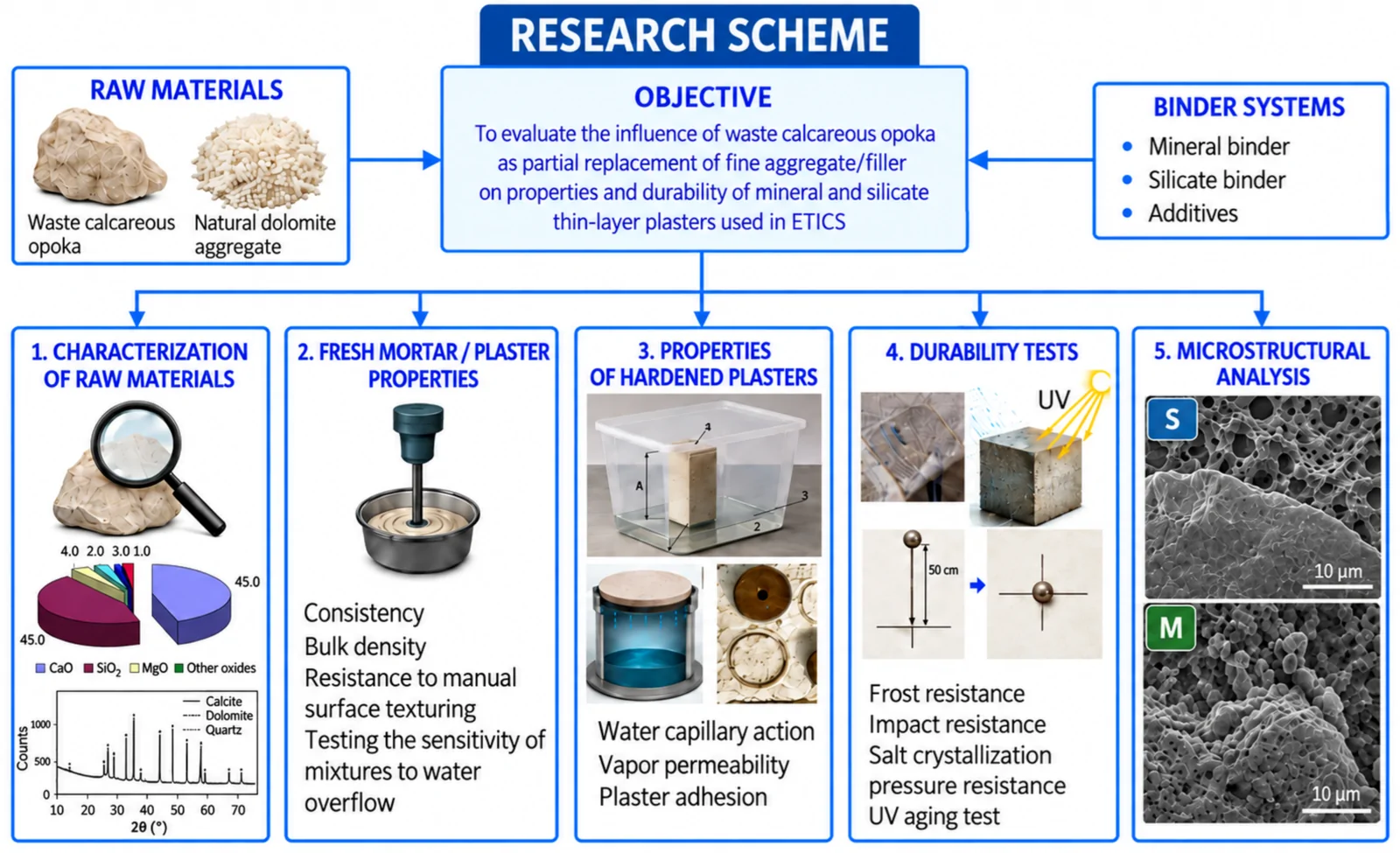
The testing programme for silicate, mineral plaster and mixtures. The arrows indicate the relationships between the investigated materials, the research objective, and the subsequent stages of the experimental programme.

The diagram (Figure 3) presents the complete testing programme for the silicate, mineral plaster and mixtures intended for use in ETICS. Detailed information on the experimental methodology, including the test procedures and measurement protocols, is provided in Supplementary File S1.

#### 2.2.1. Testing of Fresh Mixtures

(1) Bulk density

Fresh-mortar bulk density was determined in accordance with PN-EN 1015-6 [24]. A clean, dry measuring vessel of known volume V was weighed to determine its mass m_1_. The vessel was filled with fresh mortar and compacted in accordance with the standard procedure. Excess mortar was removed, the surface was struck off level with the rim of the vessel, and the filled vessel was weighed to determine m_2_. Fresh-mortar bulk density was calculated using Equation (1):

*D* = (*m*_2_ − *m*_1_)/*V* (1)

where *D* was the fresh-mortar bulk density (kg/m^3^), *m*_1_ was the mass of the empty vessel (kg), *m*_2_ was the mass of the vessel filled with fresh mortar (kg), and *V* was the vessel volume (m^3^). Three determinations were performed for each mortar.

(2) Consistency

**Figure 4:**
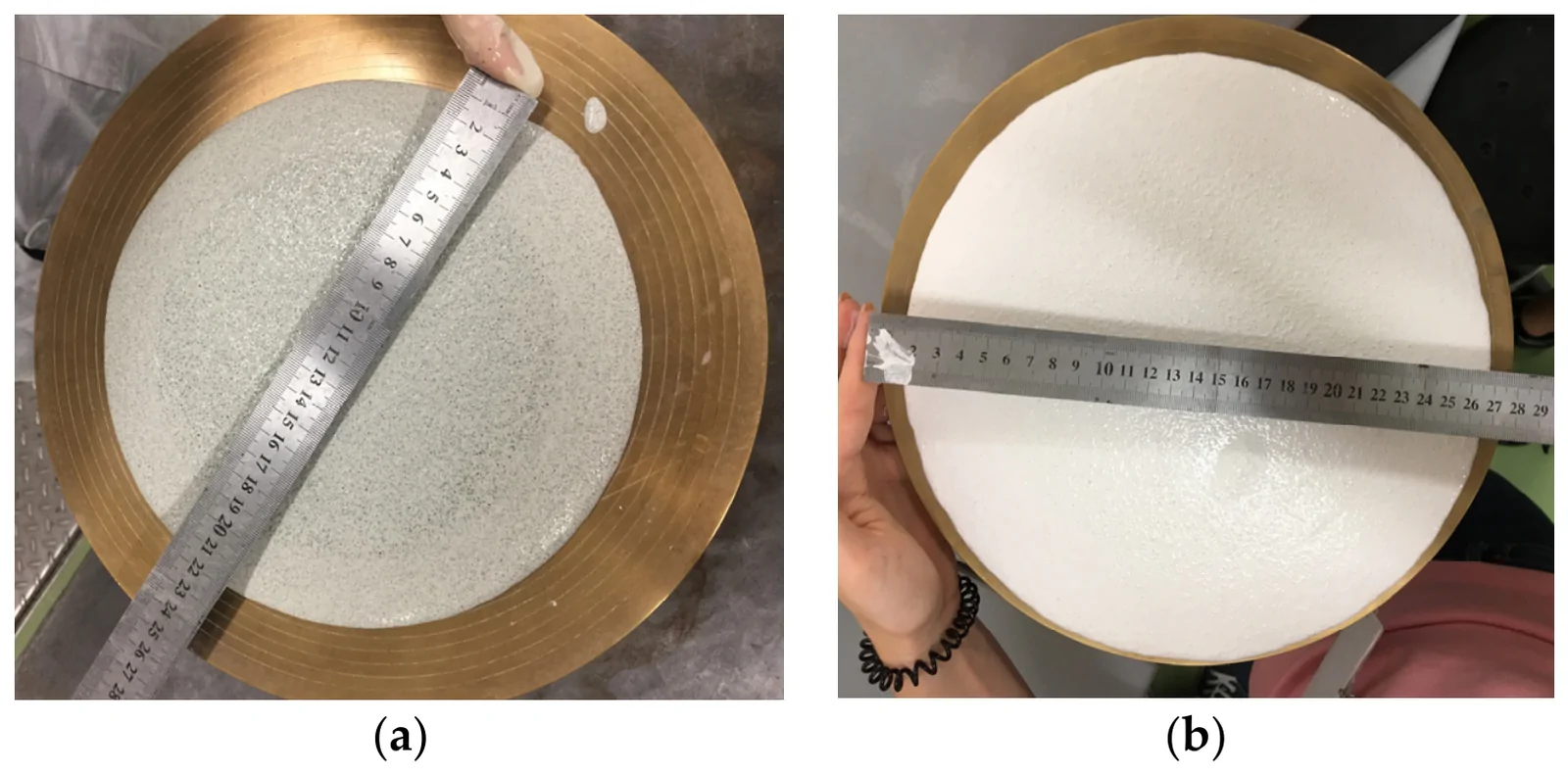
Consistency test using a flow table: (**a**) mineral plaster and (**b**) silicate plaster.

The consistency was determined in accordance with the Standard PN-EN 1015-3 [25]. The test was carried out using a flow table (Figure 4). Before each test, the disc and the edges of the mould were wiped with a damp cloth. Once dry, the surfaces were coated with a non-resinous mineral oil of very low viscosity. The mould was placed centrally on the disc of the slump table. The mortar was applied in two layers; each layer was compacted with 10 strikes of a tamper to ensure uniform filling of the mould. Excess mortar was scraped off using a flat scraper. After approximately 15 s, the mould was slowly removed, and the flow table was jolted 15 times at a constant frequency to spread the mortar across the disc. The resulting flow diameter was measured in two perpendicular directions, and the mean value was recorded. Three determinations were performed for each formulation, and the results were reported as mean ± standard deviation.

(3) Resistance during manual surface texturing

**Figure 5:**
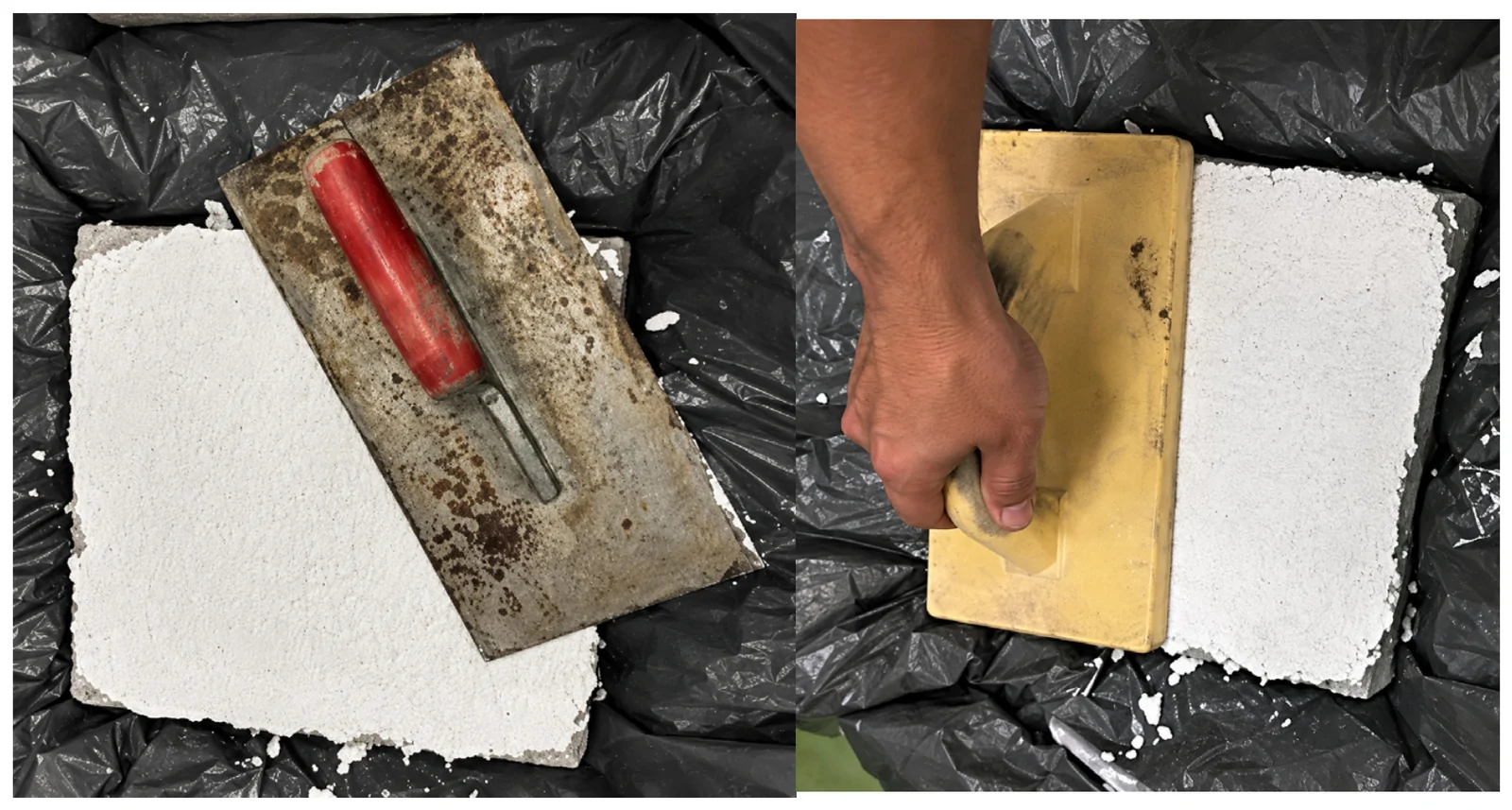
Surface resistance measurements using manual texturing.

Resistance during manual surface texturing was evaluated using an author-developed qualitative comparative procedure. The assessment was not based on a standardised test method and was intended only to compare the application-related behaviour of the investigated fresh render mixtures. Each mixture was applied to the same type of substrate using the same application procedure to obtain a visually uniform layer of comparable thickness (Figure 5). The surface was then textured using the same stainless-steel trowel. All assessments were performed by the same operator under comparable environmental conditions and at the same time after mixing.

The assessment considered resistance to trowel movement, ease of spreading and surface formation, and the perceptibility of aggregate particles beneath the tool.

(4) Mixture sensitivity to excess water

A test of the sensitivity of the mixtures to excessive water addition was carried out to assess changes in their consistency and stability following an increase in the amount of mixing water. Water was added to each mixture in stages, in quantities corresponding to a specific percentage of its mass, and the mixtures were then thoroughly mixed until homogeneous consistency was achieved.

After each stage of water addition, a slump test was carried out in accordance with the procedure used for fresh mortar. The mixture was placed in a mould on the slump table, compacted, removed from the mould, and then subjected to 15 impacts. The slump diameter was measured and an assessment was made as to whether the mixture had retained its cohesion, homogeneity and ability to form a proper texture.

A condition in which the mix exhibited excessive spread, loss of stability, segregation of components or the inability to be reliably measured was considered to exceed the acceptable limit. The test allowed a comparison of the susceptibility of individual formulations to a deterioration in workability as a result of adding an excessive amount of water. The resulting flow diameter was measured in two perpendicular directions, and the mean value was recorded. Three determinations were performed for each formulation, and the results are reported as mean ± standard deviation.

#### 2.2.2. Testing of the Hardened Plasters

(1) Capillary water absorption coefficient

The test was carried out in accordance with the requirements of the Standard PN-EN 1015-18 [26].

**Figure 6:**
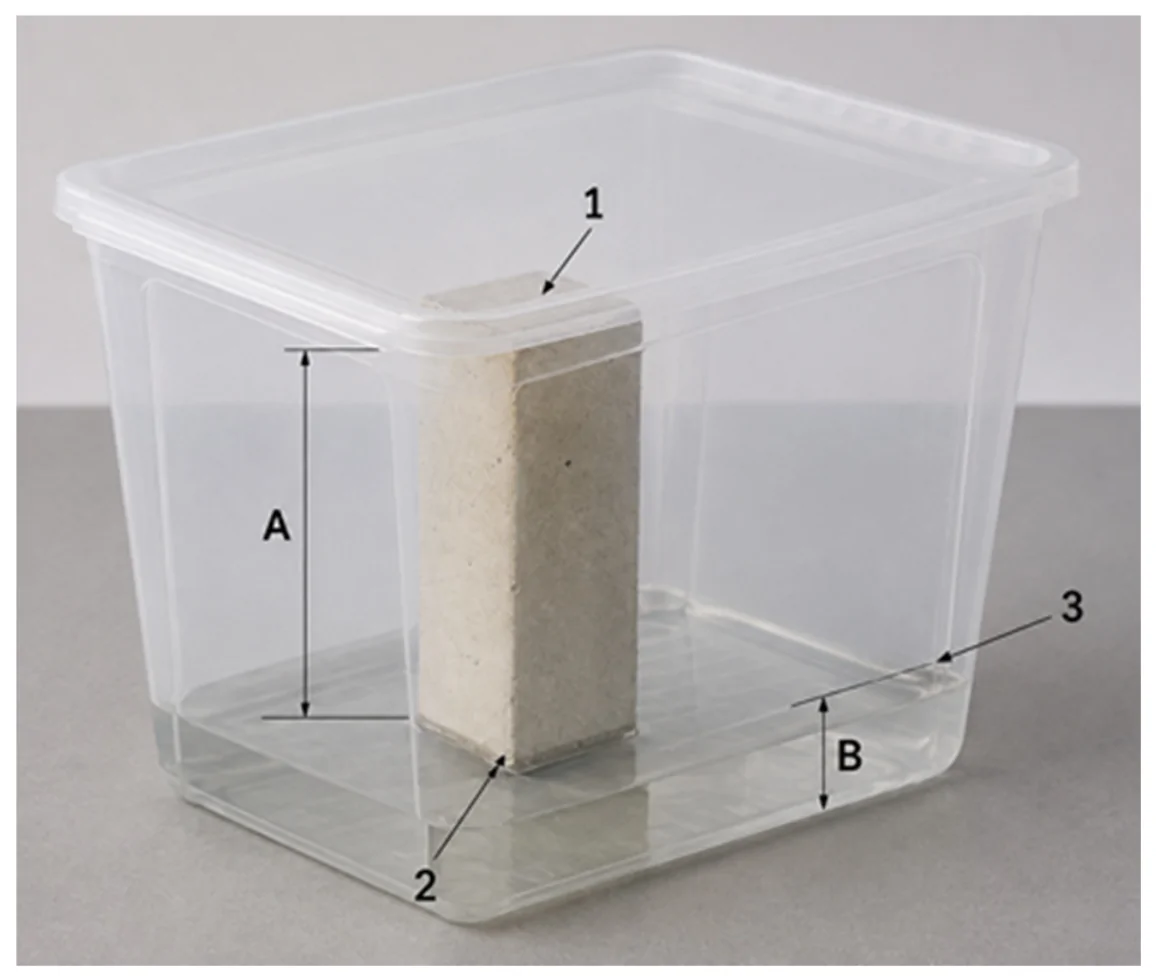
Test procedure for capillary rise in plaster. 1 is the rectangular prism specimen; 2 is the cross-sectional surface of the rectangular prism specimen; 3 is the water surface; A is approximately 80 mm; and B is the immersion depth, which is increased from 5 mm to 10 mm if the surface is very rough.

Specimens were dried to a constant mass *m*_0_ (kg) and the test face *A_s_* m^2^ was immersed by 5–10 mm (Figure 6). After 24 h, the surface water was removed and *m*_24_ (kg) was recorded. Absorption *W_A_* (kg/m^2^·24 h) was calculated as

*W_A_* = (*m*_24_ − *m*_0_)/*A_s_* (2)

Six specimens were tested, and results are reported as mean ± SD.

(2) Vapour permeability

The diffusion resistance coefficient of the plaster mortar was measured using the ‘wet cup’ method under isothermal conditions by placing the test specimen between two different environments with differing relative humidity levels in the surrounding air (PN-EN ISO 12572:2016-10 [27]). The specimens were sealed over test cups containing distilled water, and the assembled cups were placed in a desiccator under isothermal conditions. The cups were weighed at regular intervals to determine the change in mass resulting from water-vapour transmission through the specimens. The water-vapour resistance factor *μ* was determined in accordance with the calculation procedure specified in the standard. The water-vapour diffusion-equivalent air-layer thickness was calculated as

*S_d_* = *µd* (3)

where *S_d_* was the water-vapour diffusion-equivalent air-layer thickness (m), *μ* was the dimensionless water-vapour resistance factor, and *d* was the specimen thickness equal to 3 mm. Three specimens were tested for each formulation, and the results were reported as mean ± standard deviation.

(3) Freeze–thaw resistance

Freeze–thaw resistance was evaluated using a modified direct method based on PN-88/B-06250 [28]. Because free-standing thin-layer render specimens measuring 40 × 40 × 160 mm could not be prepared without altering the intended form of the material, the renders were applied to concrete blocks. Three specimens of each formulation were tested. The specimens were frozen in air at −18 ± 2 °C for at least 4 h and subsequently thawed in water at 18 ± 2 °C for 4 h. One freezing and thawing sequence constituted one cycle. Every 24 h, the specimens were visually inspected. Failure was defined as the first visually detectable crack or any partial or complete detachment of the render layer. When failure was detected, the number of completed cycles was recorded, and the specimen was removed from the test, dried to constant mass, and weighed to determine the mass loss relative to its initial dry mass. If no damage was observed, freeze–thaw cycling was continued. Owing to the 24 h inspection interval, the reported result represents the number of cycles at which damage was first detected. This modified procedure was used only for comparison of the render formulations and should not be interpreted as an assessment of the freeze–thaw resistance or service life of a complete ETICS.

(4) Resistance to the crystallisation pressure of salts

Salt-crystallisation resistance was determined using a modified procedure based on PN-EN 12370:2001 [29]. Because free-standing thin-layer render specimens could not be prepared, the renders were applied to concrete blocks. Six specimens of each formulation were tested. Mass loss was calculated relative to the initial dry mass and reported as mean ± standard deviation.

(5) Adhesion of plasters to the substrate

**Figure 7:**
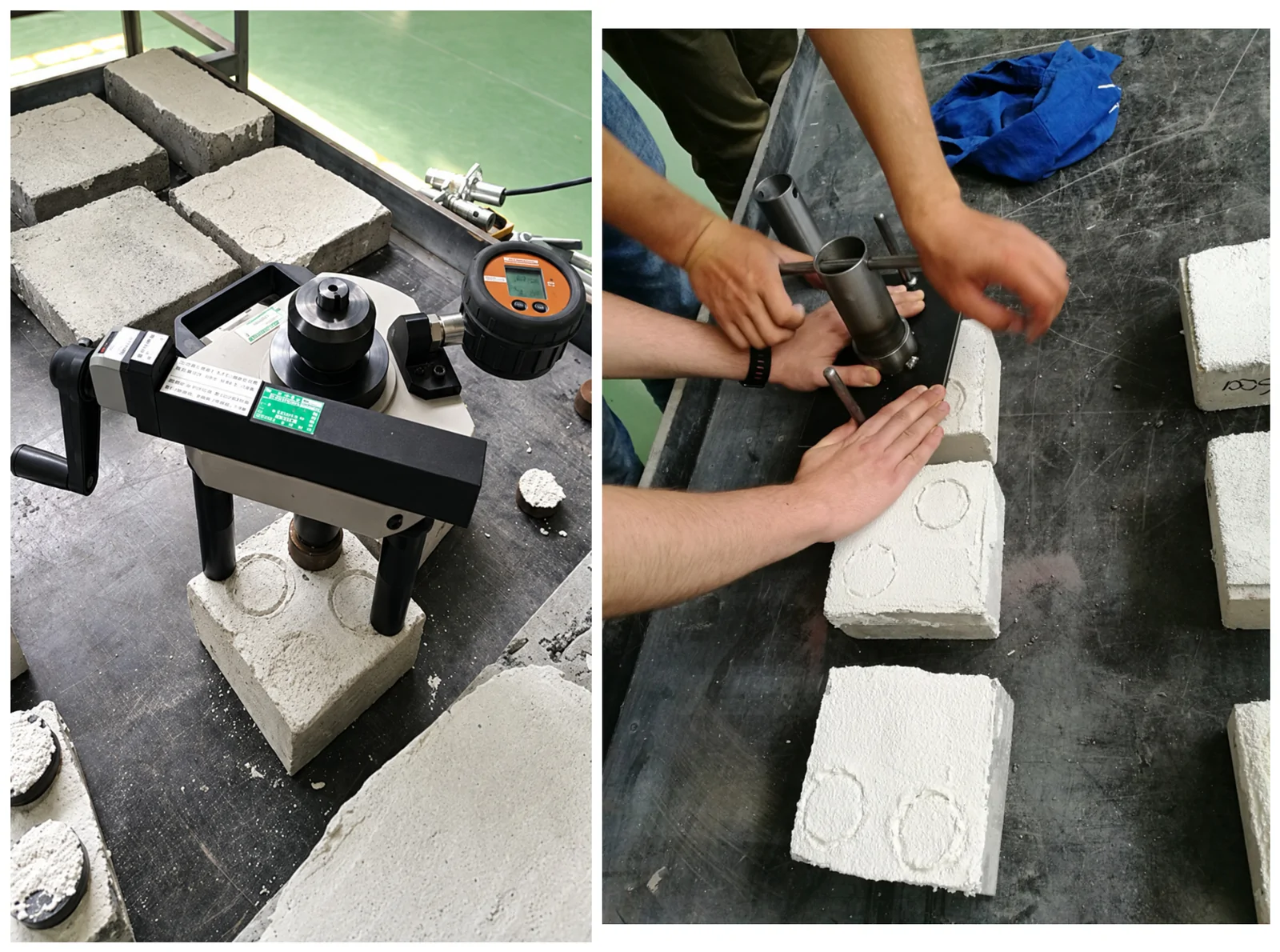
Testing the adhesion of plaster to the substrate.

The adhesion of plasters to the substrate was tested in accordance with the recommendations of Standard PN-EN 1015-12 [30]. Adhesion was defined as the maximum tensile stress induced by a peel load applied perpendicular to the surface of the mortar. The peel load was applied using a peel plate bonded to the exposed surface of the mortar under test. Five measurements were performed for each formulation, and the results were reported as mean ± standard deviation. A concrete paving block was used as the substrate. The test procedure is illustrated in Figure 7.

(6) Impact resistance of plaster

**Figure 8:**
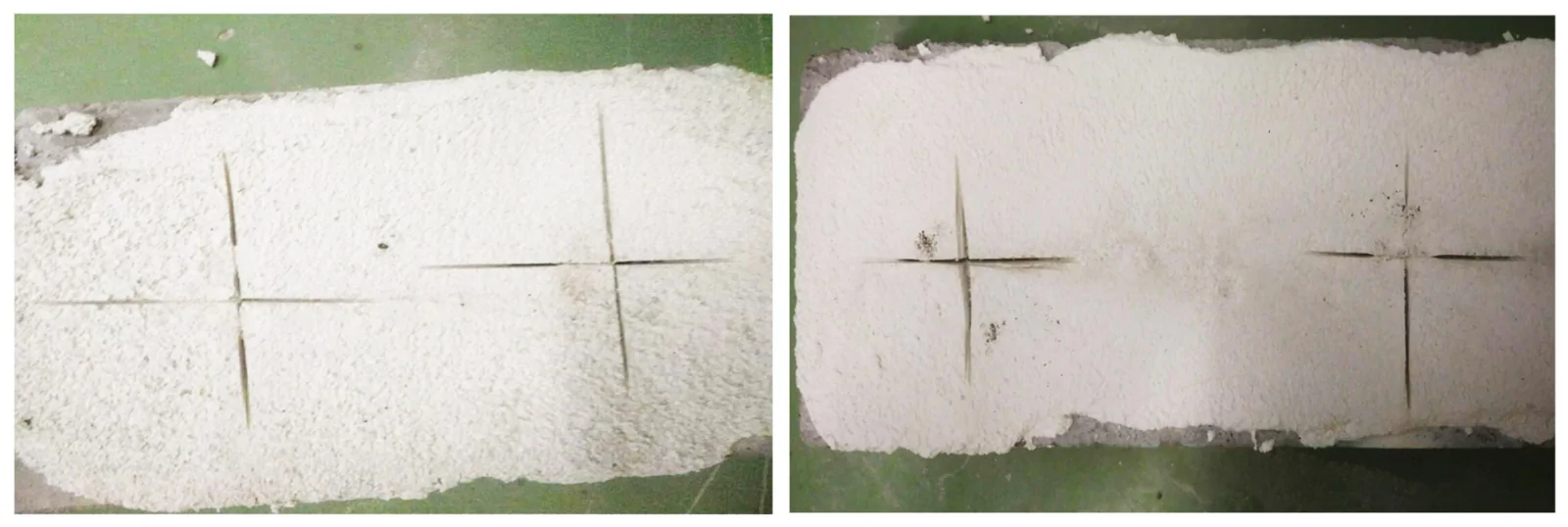
Silicate plaster after impact resistance testing.

Impact behaviour (Figure 8) was evaluated using a modified falling-ball procedure. Before impact, a cross-shaped cut was made in the render surface using an angle grinder to introduce an initial discontinuity. The experimental procedure was based on the general principle of the hard-body impact test used for rendered external thermal insulation composite systems (ETICS), as described in PN-EN 13497+A1:2021-06 [31] and in the EAD/ETAG assessment procedures for ETICS. A steel ball with a mass of 0.405 kg was then dropped vertically onto the intersection of the cuts from a height of 754 mm, corresponding to an impact energy of approximately 3 J:

E = mgh = 0.405 × 9.81 × 0.754 = 3.0 J(4)

where E was the impact energy, m was the ball mass, g was the gravitational acceleration, and h was the drop height.

Therefore, the paper does not present the procedure as a standard test and does not assign the investigated materials to standard ETICS impact-resistance categories. Before impact, a cross-shaped cut was introduced into the surface of each render using an angle grinder (Figure 8). The steel ball was then dropped onto the intersection of the cuts. The cut acted as an artificially introduced initial discontinuity and enabled the behaviour of the render in the presence of existing damage to be evaluated. In contrast to a standard impact applied to an intact surface, the procedure was primarily intended to examine the subsequent development of damage rather than its initial formation.

After impact, the specimens were visually examined for crack extension from the ends of the cuts, local spalling, and partial or complete detachment of the render layer from the substrate.

(7) UV ageing test

The resistance of the coatings to UV-induced degradation was evaluated using a TestAn Xentest 2200 xenon-arc weathering chamber (TestAn, Gdańsk, Poland)**.** The exposure conditions were selected to reproduce, in an accelerated manner, the effect of approximately one year of outdoor solar radiation under climatic conditions representative of Poland. The adopted test parameters were as follows: total exposure time of 720 h, chamber temperature of 63 °C and irradiance of 0.51 W/m^2^. The results were expressed as the change in capillary water absorption after ageing relative to the initial value and as the reduction in adhesion strength. A positive value of the index indicates an increased susceptibility of the render to liquid-water transport and, consequently, a deterioration in its resistance to moisture exposure. The change in capillary water absorption was calculated according to the following equation:

Δ*A* = [(*A_after_* − *A_before_*)/*A_before_*] × 100% (5)

where *A_befor_*_e_ was the capillary water absorption measured before ageing, kg/(m^2^·24 h) and *A_afte_*_r_ was the capillary water absorption measured after ageing, kg/(m^2^·24 h).

The reduction in adhesion strength was determined as the relative decrease in bond strength Δ*f_adh_* between the render and the substrate:

Δ*f_adh_* = [(*f_before_* − *f_after_*)/*f_before_*] × 100% (6)

where *f_before_* was the adhesion strength measured before ageing, N/mm^2^, and *f_after_* was the adhesion strength measured after ageing, N/mm^2^.

The greater the value of Δ*f_a__dh_*, the more pronounced the degradation of the render–substrate interface resulting from exposure to UV radiation, cyclic wetting and drying, and moisture fluctuations.

(8) Microstructure—scanning microscopy

The observations were carried out using a Quanta 250 FEG microscope (FEI, Hillsboro, OR, USA) equipped with an EDAX energy-dispersive X-ray spectroscopy (EDS) system for chemical composition analysis. The SEM samples, in the form of fragments, were mounted on a carbon holder using carbon adhesive. The specimens were then sputter-coated with a layer of carbon approximately 50 nm thick in a sputtering chamber to achieve surface conductivity. Two magnifications were used: 500× and 5000×.

#### 2.2.3. Data Analysis

(1) Multi-criteria analysis and Pareto analysis

Seven quantitative parameters representing the key aspects of hardened-render performance were selected for the analysis: capillary water absorption coefficient, water-vapour diffusion-equivalent air-layer thickness *S_d_*, the number of freeze–thaw cycles to failure, mass loss after freeze–thaw testing, the number of sodium sulphate crystallisation cycles, mass loss after salt-crystallisation testing, and adhesion strength to the substrate.

Normalisation was performed separately for the two matrix types. This approach enabled the effect of the WCOR aggregate replacement level to be evaluated within each render type, without the analysis being dominated by the fundamental differences arising from the use of different binder systems.

For benefit criteria, in which higher values represented more favourable performance, the normalised value was calculated using Equation (7):

*N_i_* = (*x_i_* − *x_min_*)/(*x_max_* − *x_min_*) (7)

where $N_{i}$ was the normalised value of a given criterion for formulation ii, $x_{i}$ was the measured value for that formulation, and *x_min_* and *x_max_* were, respectively, the minimum and maximum values of the criterion within the corresponding render series. Equation (7) was applied to the number of freeze–thaw cycles to first visible damage, the number of salt-crystallisation cycles to first visible damage, and adhesion strength.

For cost criteria, in which lower values represented more favourable performance, inverse normalisation was applied according to Equation (8):

*N_i_* = (*x_max_* − *x_i_*)/(*x_max_* − *x_min_*) (8)

In each case, the normalised value therefore ranged from 0 to 1, where 1 represented the most favourable performance within the analysed group of renders, while 0 corresponded to the least favourable performance.

Subsequently, an overall performance index (*PI*) was calculated as the arithmetic mean of the seven normalised criteria:

(9) $$PI_{i}=\;\sum_{j=1}^{n}w_{j}N_{ij},\;\;\;\;\;\;\sum_{j=1}^{n}w_{j}=1,\;\;\;\;\;\;\;n=7$$

where *PI* was the performance index of formulation *i*, *N_ij_* was the normalised value of criterion j for formulation i, $w_{j}$ was the weight assigned to criterion *j*, and *n* was the number of criteria. Because equal weighting did not necessarily reflect the relative engineering importance of the individual performance criteria, the stability of the resulting ranking was subsequently evaluated using alternative weighting scenarios. The performance index should therefore be interpreted as a comparative tool for assessing trade-offs among the investigated formulations within each render family, rather than as an absolute measure of material quality.

(2) Application-related constraint and weighting sensitivity

The resistance encountered during manual texturing was not incorporated directly into the calculation of the PI because it was assessed using a three-level ordinal scale rather than as a continuous physical quantity. For both render families, this parameter increased from 1 for the reference formulation, through 2 for the intermediate variants, to 3 for the formulation containing 100% WCOR replacement. The preceding analysis further indicated that a moderate level of texturing resistance may have been beneficial for maintaining the applied surface texture, whereas an excessive increase in resistance at the highest WCOR content may have hindered material application.

Accordingly, this parameter was used as an additional practical criterion in the interpretation of the ranking. Classes 1–2 were considered to represent the most favourable application range, whereas class 3 was treated as an indication of the reduced ease of texturing. This approach made it possible to distinguish between the formulation providing maximum durability and the one offering the most balanced overall performance.

The normalised results were subsequently presented in the form of radar charts and a multi-criteria ranking, enabling direct identification of the trade-offs associated with increasing WCOR content and the differentiation between an optimum based on maximum durability and an optimum providing the most balanced combination of performance characteristics.

(3) Pareto analysis and graphical presentation

Pareto analysis was used to identify non-dominated formulations, i.e., formulations for which improvement in one performance criterion would require deterioration in at least one other criterion. The full analysis considered all seven quantitative criteria. A reduced two-dimensional projection was additionally prepared using capillary water absorption as the criterion to be minimised and freeze–thaw resistance as the criterion to be maximised. The number of salt-crystallisation cycles was represented by marker size, whereas adhesion strength was represented by marker outline width. Radar charts and a multi-parameter performance map were used as supplementary graphical tools for visualising the trade-offs among the investigated properties.

## 3. Results and Discussion

### 3.1. Properties of Fresh Mixtures

**Figure 9:**
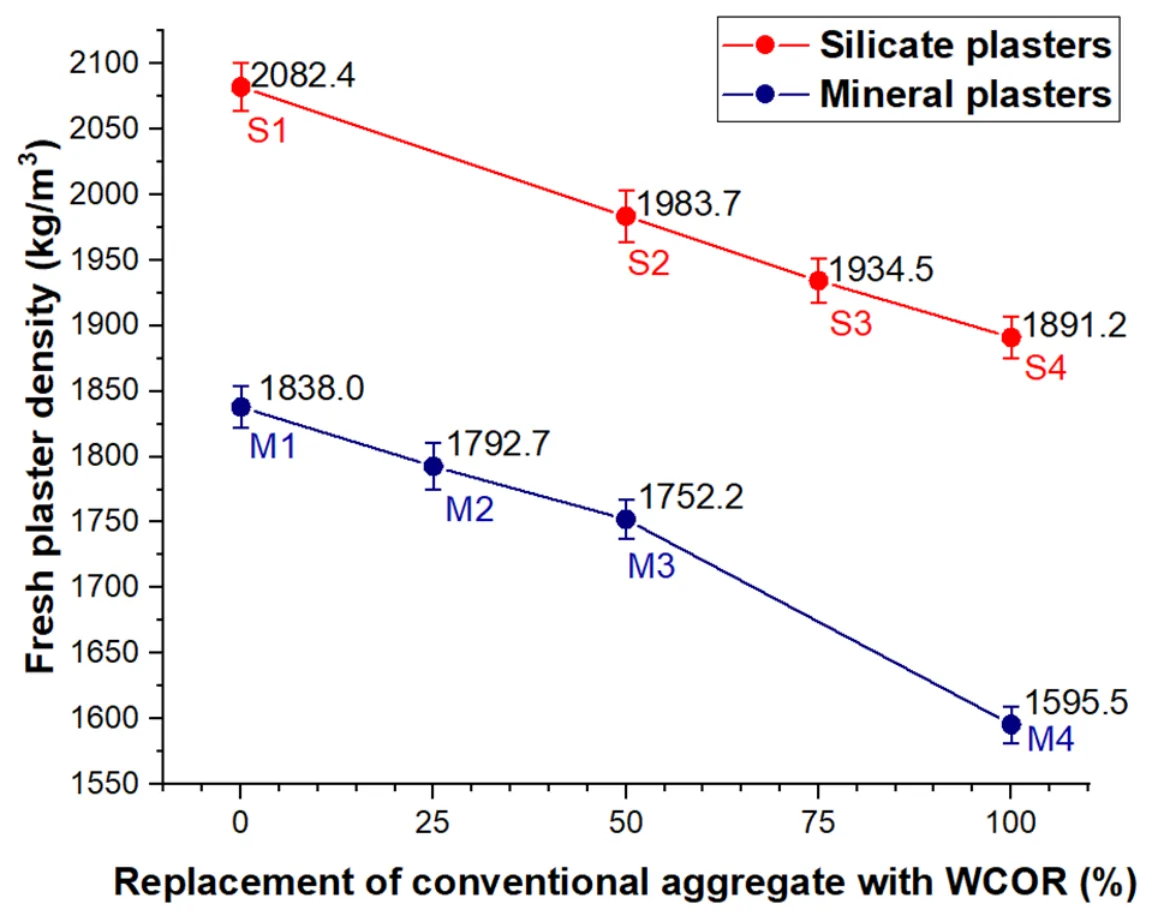
Density of fresh mixtures.

**Figure 10:**
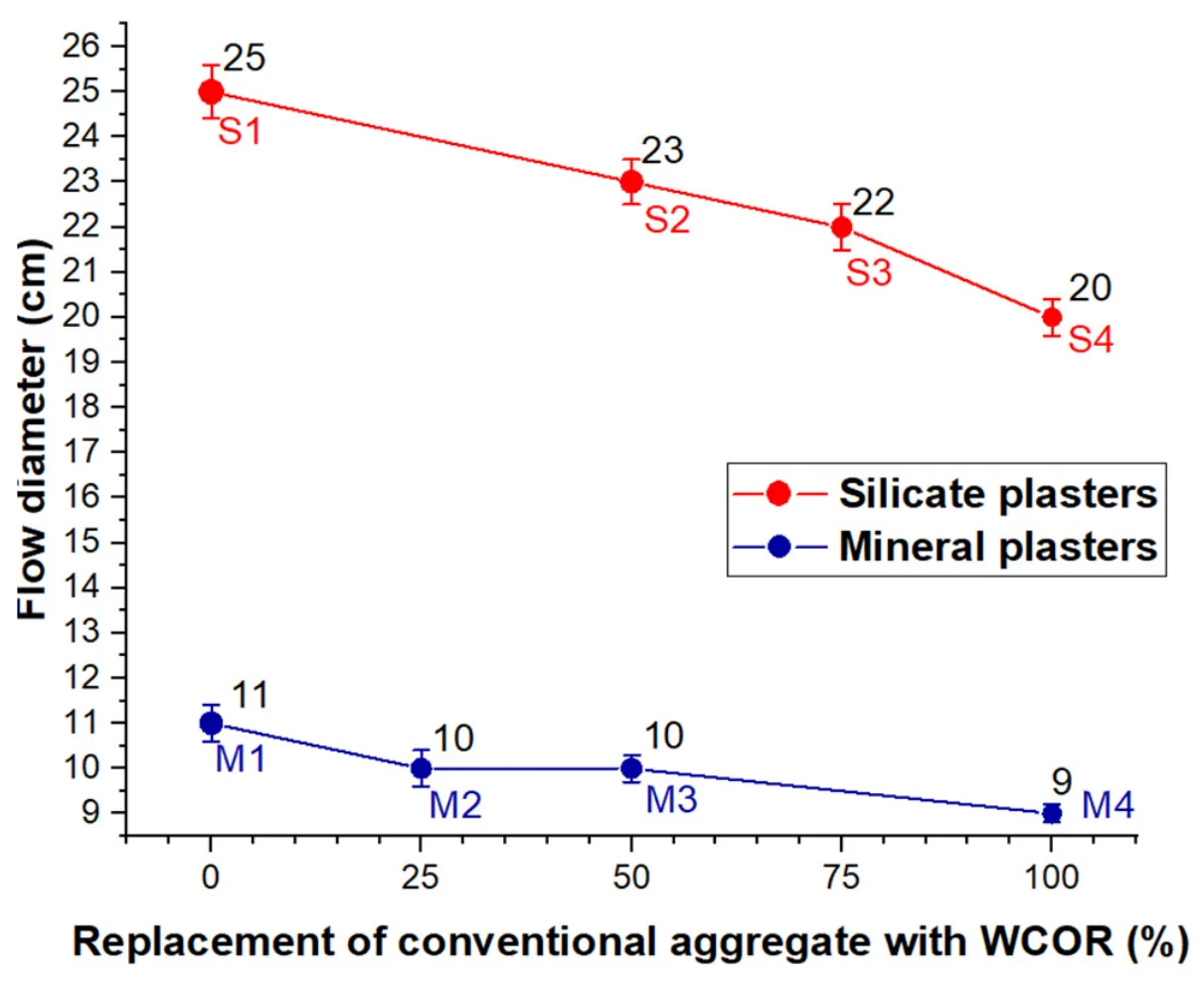
Consistency of fresh mixtures.

The properties of the render mixtures are presented in Figure 9 and Figure 10 and Table 4.

Increasing the WCOR replacement level systematically reduced the fresh bulk density of both render families. For the silicate renders, density decreased from 2082.4 kg/m^3^ for S1 to 1963.7, 1934.5, and 1891.2 kg/m^3^ for S2–S4, corresponding to reductions of 5.7%, 7.1%, and 9.2%, respectively. For the mineral renders, it decreased from 1838.0 kg/m^3^ for M1 to 1792.7, 1752.2, and 1595.5 kg/m^3^ for M2–M4, representing reductions of 2.5%, 4.7%, and 13.2%. This reduction resulted from replacing dense dolomite with more porous and heterogeneous WCOR, which may have increased the volume of interparticle voids and the retention of water and air in the fresh mixture [32,33]. Similar density reductions have been reported for mortars containing porous recycled aggregates [33]. The particularly pronounced reduction for M4 was consistent with the larger mass proportion of replaced aggregate: approximately 50% of the total mixture mass compared with 39% for S4.

Increasing WCOR content also progressively reduced the flow diameter. For the silicate renders, it decreased from 25 cm for S1 to 23, 22, and 20 cm for S2–S4, respectively. The mineral renders exhibited lower values, decreasing from 11 cm for M1 to 10 cm for M2 and M3 and 9 cm for M4.

The decreasing flow diameter indicates reduced workability and a greater resistance to flow. A similar response was reported for lime–cement mortars containing ground calcareous opoka and was attributed to the rough surface, high specific surface area, and water demand of the opoka particles [34]. Dhananjaya et al. [35] likewise found that stone-processing waste reduced the workability of rendering mortars and increased their water demand.

Table 4 shows the resistance of fresh plaster mixes to manual texturing of the surface and their sensitive reaction to additional water, as evidenced by an increase in the flow diameter.

Increasing opoka content increased resistance during manual surface texturing, from score 1 for the reference mixtures S1 and M1 to score 3 for the fully substituted mixtures S4 and M4. This trend corresponded to the reduced flow diameter and was attributed to the irregular, rough, and porous morphology of WCOR particles, which increased internal friction and reduced mixture workability [36].

The addition of 2.5% of water affected the two render types differently. Silicate renders showed only a slight increase in flow diameter, by approximately 4–10%, whereas mineral renders were much more sensitive, with increases of about 100–120%. After adding 7.5% of water, the flow diameter reached approximately 27–29 cm for both render types.

The pronounced response of the mineral mixtures to additional water may indicate that their initial water content was close to the amount required to establish a continuous liquid phase between the particles. Once the water necessary to fill the interparticle voids is exceeded, additional water can form a continuous film on the particle surfaces. This film reduces direct particle-to-particle contact and friction within the granular skeleton, thereby increasing flowability [37,38]. A similar behaviour has been reported for rendering and lime-based mortars, in which relatively small additions of water produced substantial changes in fresh-state flow when the initial water content was close to this threshold [37,39].

The lower sensitivity of silicate renders to additional water may be associated with the presence of water-retaining and rheology-modifying constituents. In particular, methylcellulose can increase cohesion and water retention, thereby limiting the mobility of the liquid phase and reducing changes in flowability following water addition [40]. Previous studies have similarly shown that cellulose ethers increase water retention and reduce the flowability of rendering mortars [41]. The lower sensitivity of silicate renders to additional water may be attributed to the combined action of the styrene–acrylic dispersion, cellulose ether, dispersing agent, and polyurethane thickener, which enhanced water retention, cohesion, and mixture stability [40]. Consequently, part of the added water was retained within the polymer-modified matrix rather than immediately increasing flowability.

The increase in manual-texturing resistance with decreasing flow diameter can therefore be related to reduced free-water content and increased interparticle friction. Moderate resistance may improve texture retention, whereas excessive resistance at high WCOR contents can impair workability and surface uniformity.

### 3.2. Properties of Hardened Renders

#### 3.2.1. Capillary Water Absorption Coefficient

**Figure 11:**
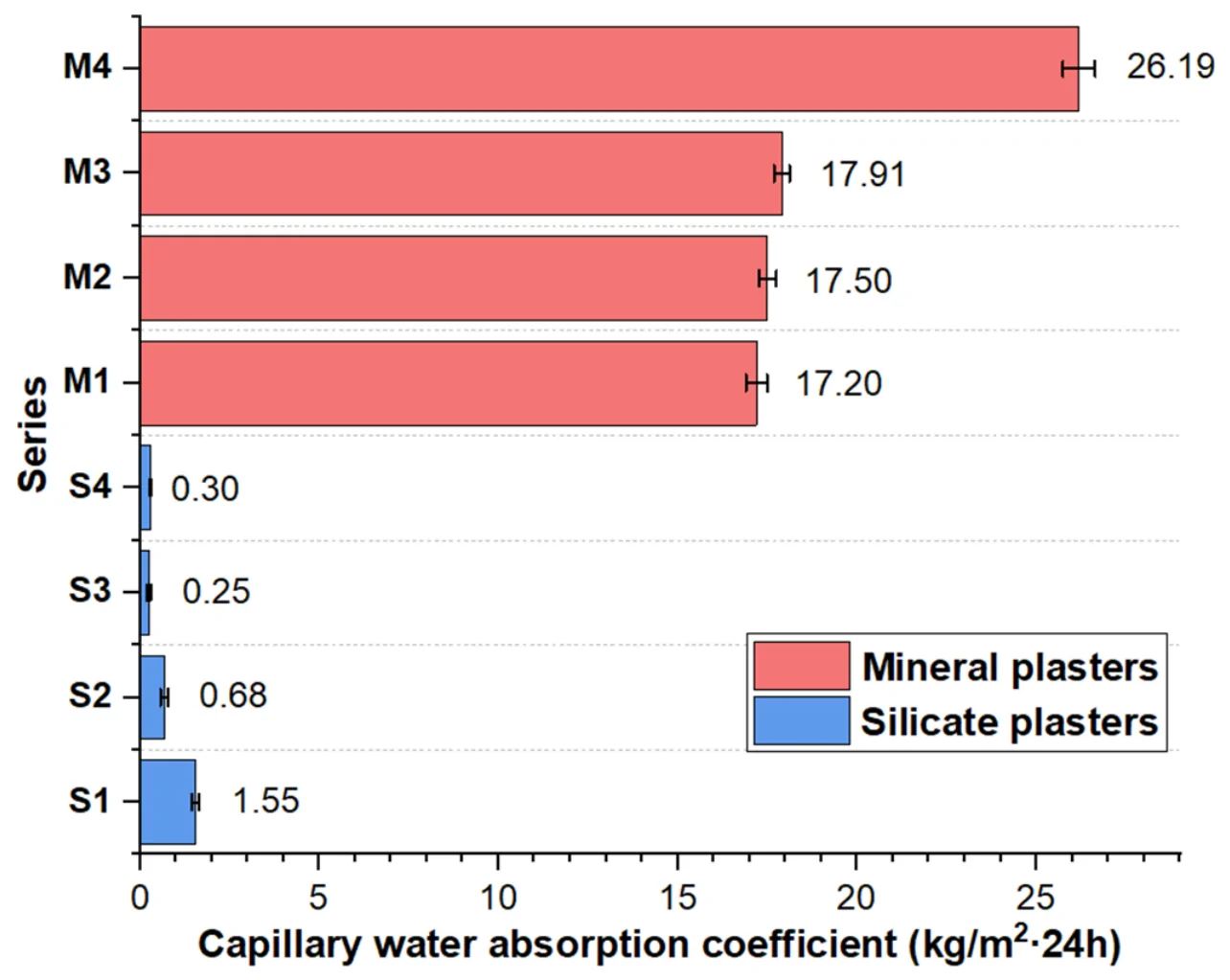
Capillary water absorption coefficients of tested plasters.

The moisture-related properties of the renders were evaluated in terms of their ability to transport liquid water and water vapour. Figure 11 presents the capillary water absorption coefficient.

The capillary water absorption coefficient depended strongly on both the render type and the WCOR replacement level, with silicate renders showing substantially lower water uptake than mineral renders.

For the silicate series, the coefficient decreased from 1.55 kg/(m^2^·24 h) for S1 to 0.68 and 0.25 kg/(m^2^·24 h) for S2 and S3, corresponding to reductions of approximately 56% and 84%, respectively. A slight increase to 0.30 kg/(m^2^·24 h) was observed for S4, although this value remained about 81% lower than that of S1. Thus, the lowest capillary absorption was obtained for S3 with 75% WCOR replacement, while complete replacement provided no further improvement. A different trend was observed for the mineral renders. The coefficients for M1, M2, and M3 were similar, at 17.2, 17.6, and 17.9 kg/(m^2^·24 h), respectively. In contrast, M4 showed a pronounced increase to 26.2 kg/(m^2^·24 h), approximately 52% above the reference value.

These results demonstrate that the influence of WCOR on capillary water transport was strongly matrix-dependent. The effect cannot be attributed solely to aggregate porosity, as moisture transport is also governed by pore-size distribution and connectivity [42], as well as by the binder composition and resulting microstructure. In the silicate renders, polymeric and hydrophobic components may have limited the formation of continuous capillary pathways, whereas complete WCOR replacement in the mineral render promoted substantially greater water uptake.

The low capillary water absorption coefficients observed for the silicate renders can be attributed to the combined action of several mechanisms affecting pore structure and pore-wall wettability, as supported by previous studies on polymer dispersions, hydrophobic additives, and microstructural modifiers [43,44].

The styrene–acrylic dispersion may reduce capillary water transport by modifying pore geometry and connectivity, even when total porosity increases. Previous studies reported reductions in sorptivity of about 36% [43] and in capillary water absorption of more than 55% when capillary pore connectivity was reduced below 11% [45].

Silane- and polysiloxane-based additives have been shown to increase the water contact angle and improve resistance to liquid-water penetration [46,47,48,49]. In addition, methylcellulose and other stabilising additives can influence the pore structure and continuity of transport pathways within the matrix [50,51]. Mineral fillers may also increase the tortuosity of the pore network, thereby limiting the formation of continuous capillary paths and reducing water transport [52].

#### 3.2.2. Vapour Permeability

**Figure 12:**
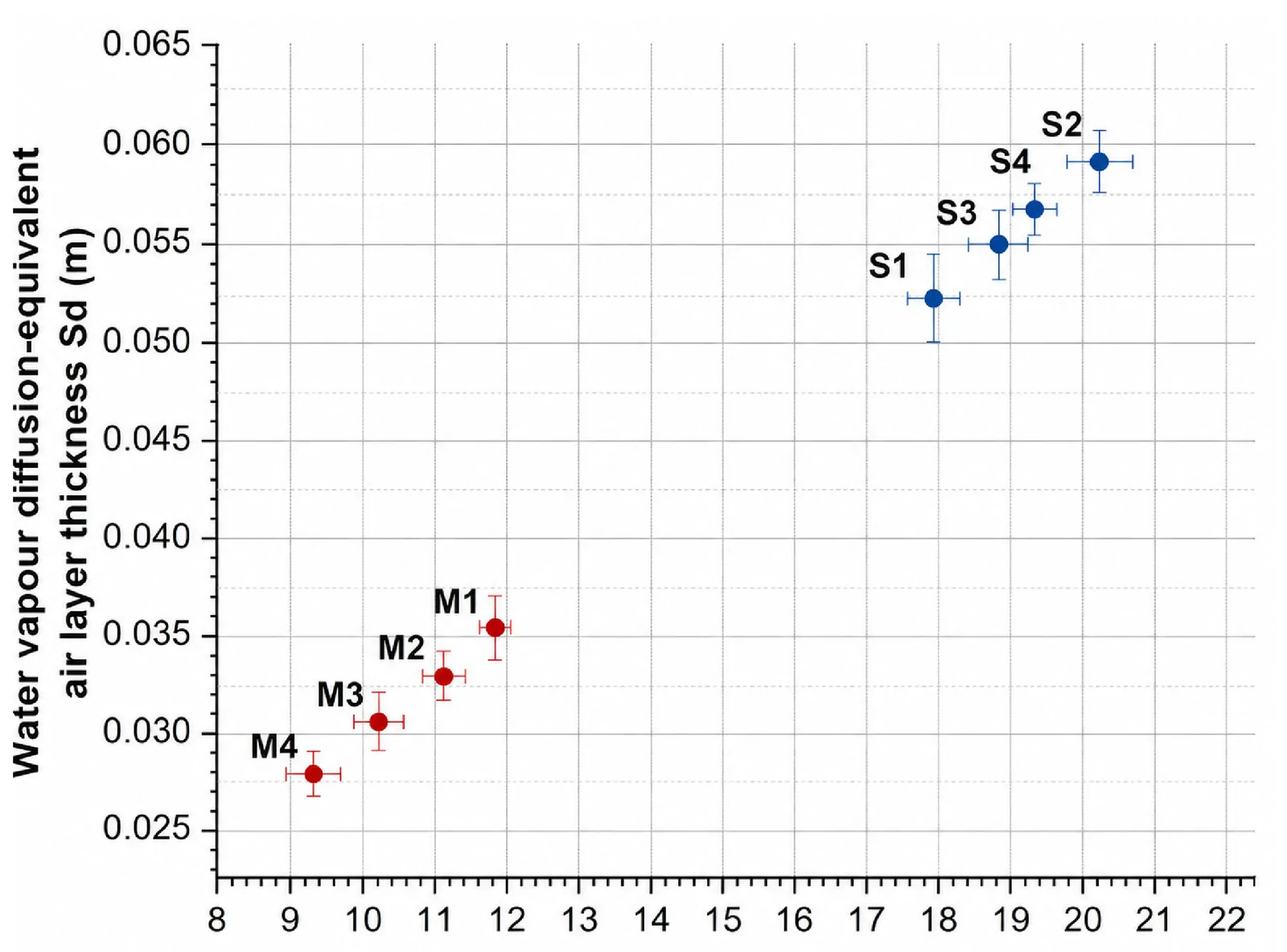
Comparison of the water-vapour diffusion resistance factor μ and the water-vapour diffusion-equivalent air-layer thickness Sd for the investigated silicate and mineral renders.

Figure 12 presents the values of the water-vapour diffusion resistance factor together with the corresponding water-vapour diffusion-equivalent air-layer thicknesses.

The test results showed that the silicate and mineral renders differed in terms of water-vapour diffusion resistance. The silicate renders exhibited higher values of the water-vapour diffusion resistance factor, µ, ranging from 17.4 to 19.7, whereas for the mineral renders the values ranged from 9.3 to 11.8. Within the silicate series, partial replacement of the dolomite aggregate with WCOR initially increased diffusion resistance, with the maximum value recorded for S3. An opposite trend was observed for the mineral renders, where increasing the WCOR content resulted in a gradual decrease in µ, indicating improved water-vapour permeability.

These differences confirm that the effect of the waste aggregate depended on the type of render matrix and the resulting pore structure. The Sd values varied proportionally with µ, since they were determined for a constant render-layer thickness of approximately 3 mm.

#### 3.2.3. Freeze–Thaw Resistance

**Figure 13:**
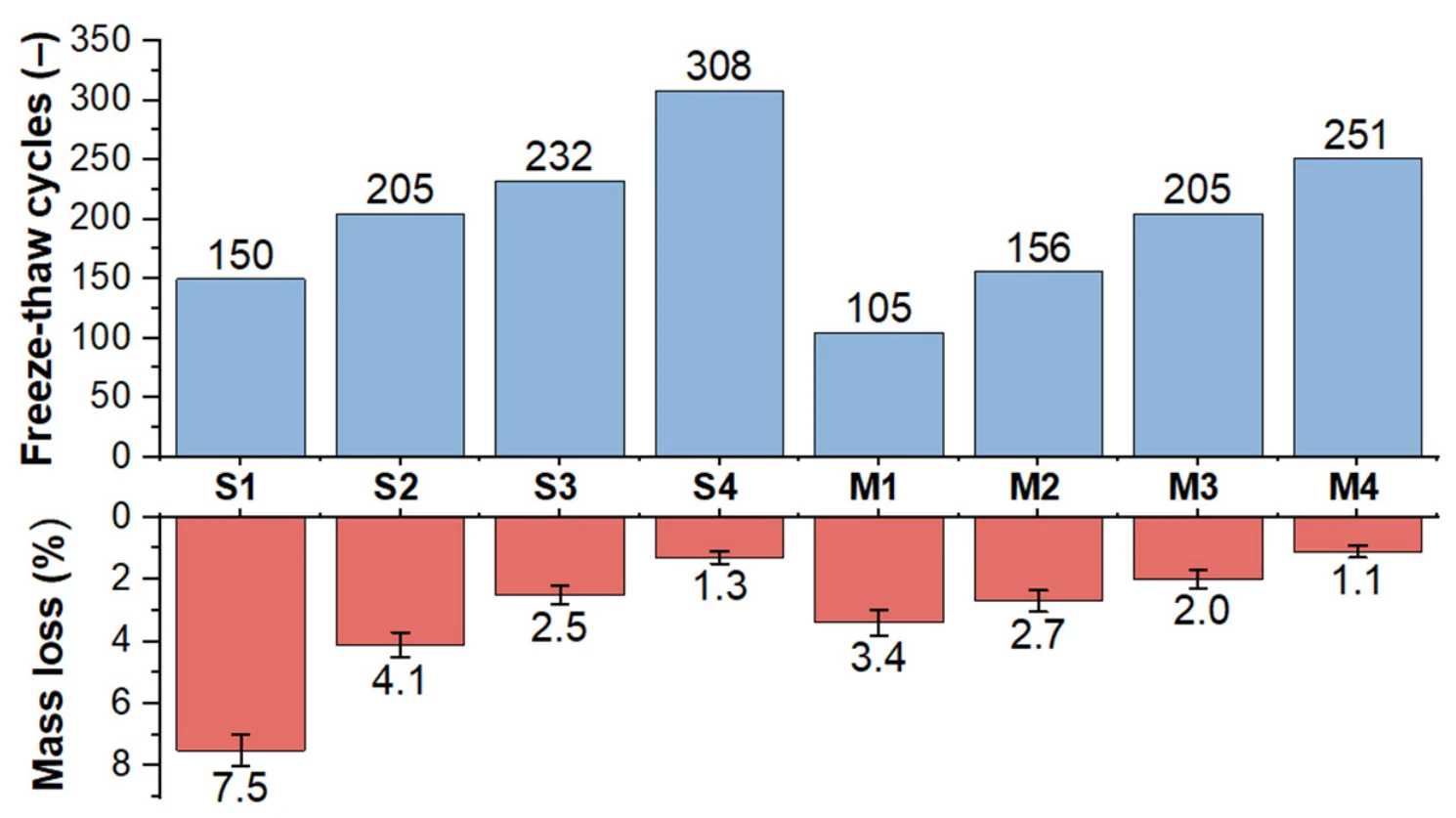
Freeze–thaw resistance of the investigated renders.

The freeze–thaw resistance results are presented in Figure 13. The renders were subjected to repeated freezing and thawing cycles, and the test was continued until material failure occurred.

**Figure 14:**
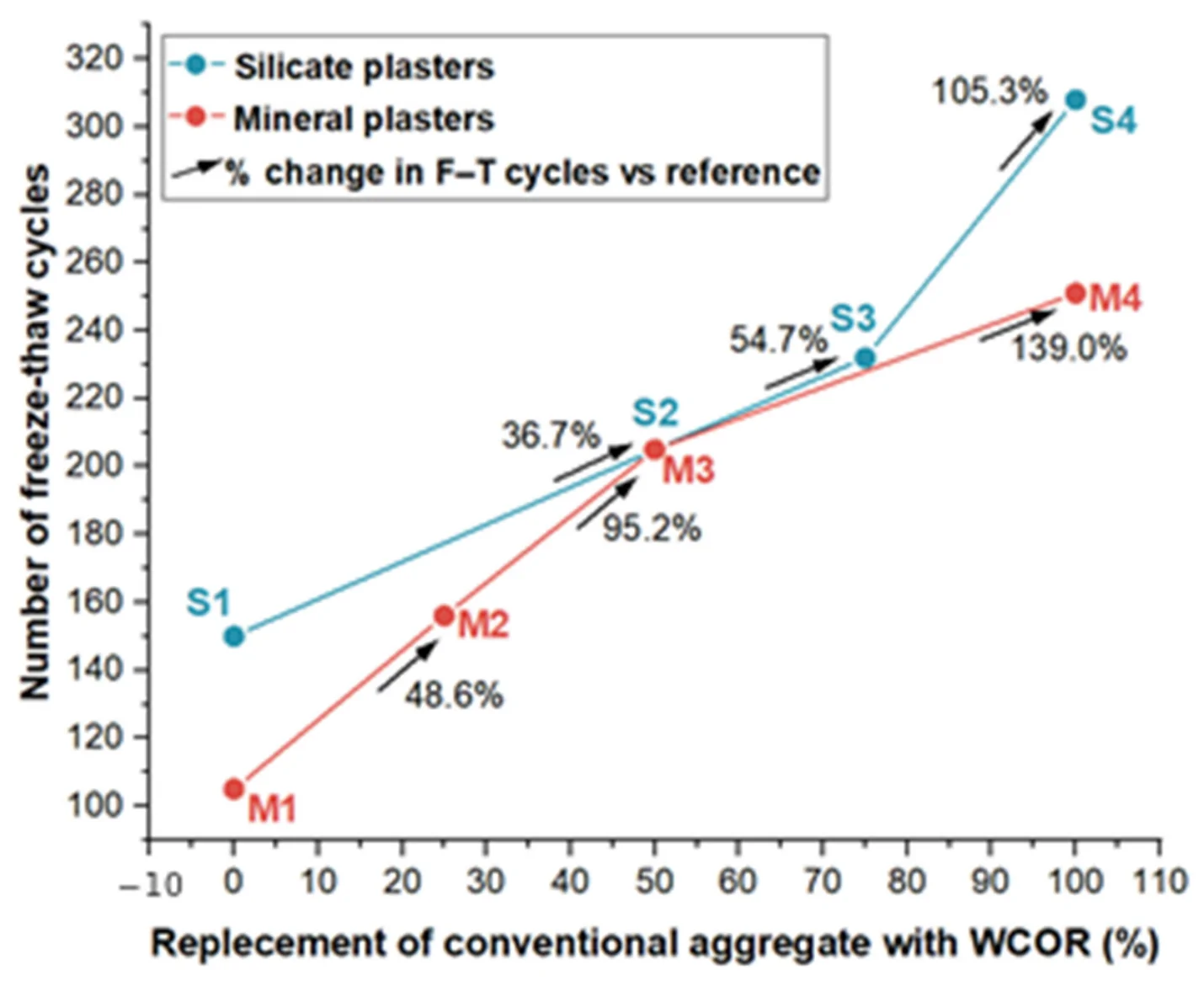
Percentage increase in freeze–thaw cycles relative to the reference renders.

The percentage changes in the number of freeze–thaw (F–T) cycles to failure with increasing aggregate replacement level, calculated relative to the corresponding reference samples (S1 and M1), are presented in Figure 14.

Increasing the WCOR replacement level consistently improved the freeze–thaw durability of both silicate and mineral renders. In the silicate series, the number of cycles to failure increased from 150 for S1 to 308 for S4, while mass loss decreased from 7.5% to 1.3%. This approximately corresponds to a 105% increase in the number of cycles and an 83% reduction in mass loss. A similar trend was observed for the mineral renders, where the number of cycles increased from 105 for M1 to 251 for M4 and mass loss decreased from 3.4% to 1.1%, corresponding to improvements of approximately 139% and 68%, respectively. The intermediate replacement levels showed a progressive improvement in both parameters.

Overall, the silicate renders exhibited higher freeze–thaw resistance than the corresponding mineral renders. Nevertheless, in both groups, complete replacement of dolomite aggregate with WCOR resulted in the highest number of cycles to failure and the lowest mass loss.

The beneficial effect of WCOR may be related to changes in pore structure and the formation of additional voids capable of accommodating stresses generated during ice formation. Freeze–thaw resistance depends not only on total porosity but also on pore-size distribution, shape, and connectivity [53,54]. In silicate renders, polymer and polysiloxane additives may additionally reduce the degree of water saturation and the amount of freezable water, thereby limiting hydraulic pressure and microcrack development [48,55]. In mineral renders, the improvement observed despite relatively high capillary absorption suggests that pressure-relief pores and water redistribution within the pore system played a more important role than capillary uptake alone.

#### 3.2.4. Resistance to the Crystallisation Pressure of Salts

**Figure 15:**
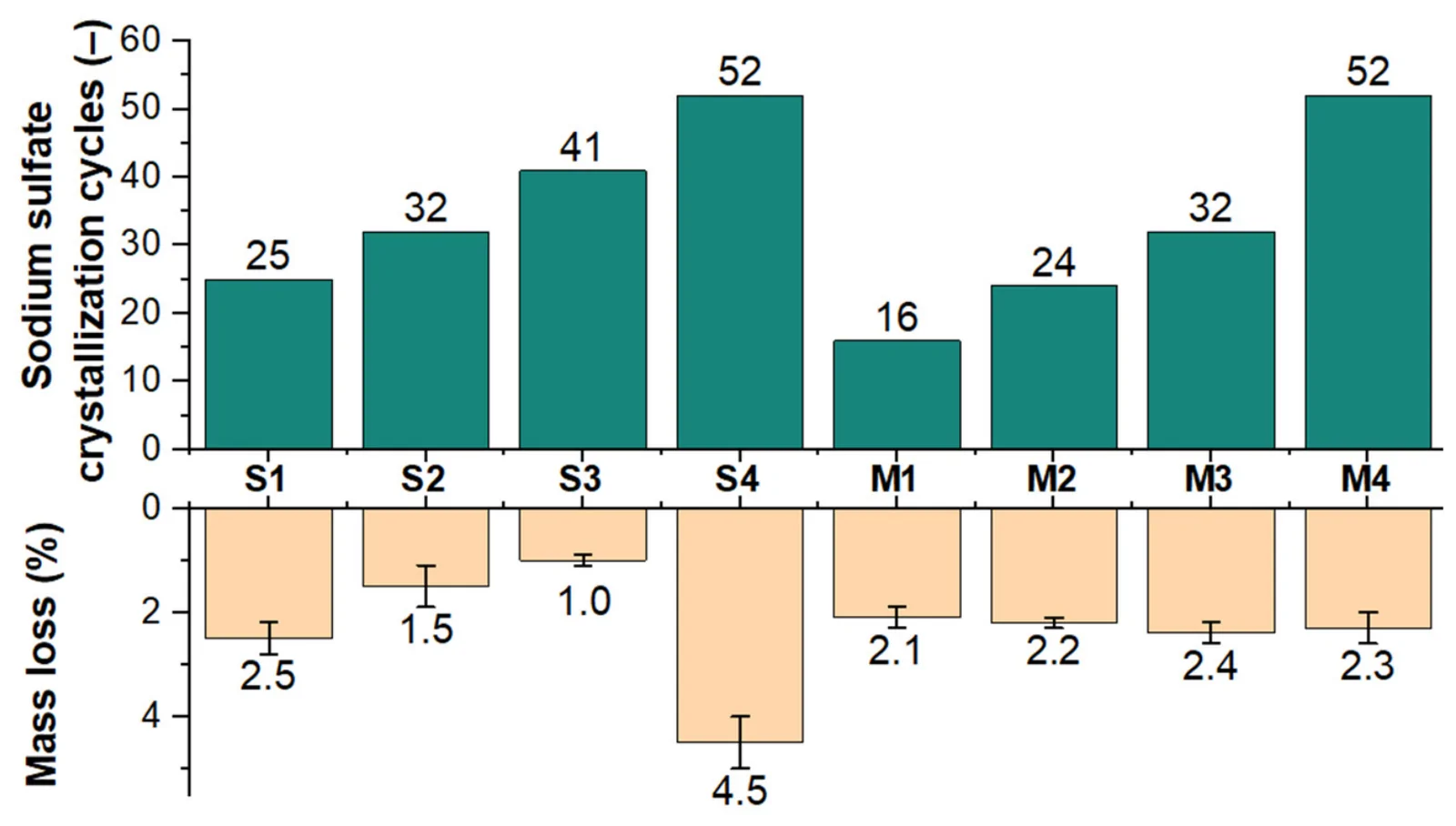
Results of resistance to salt crystallisation.

**Figure 16:**
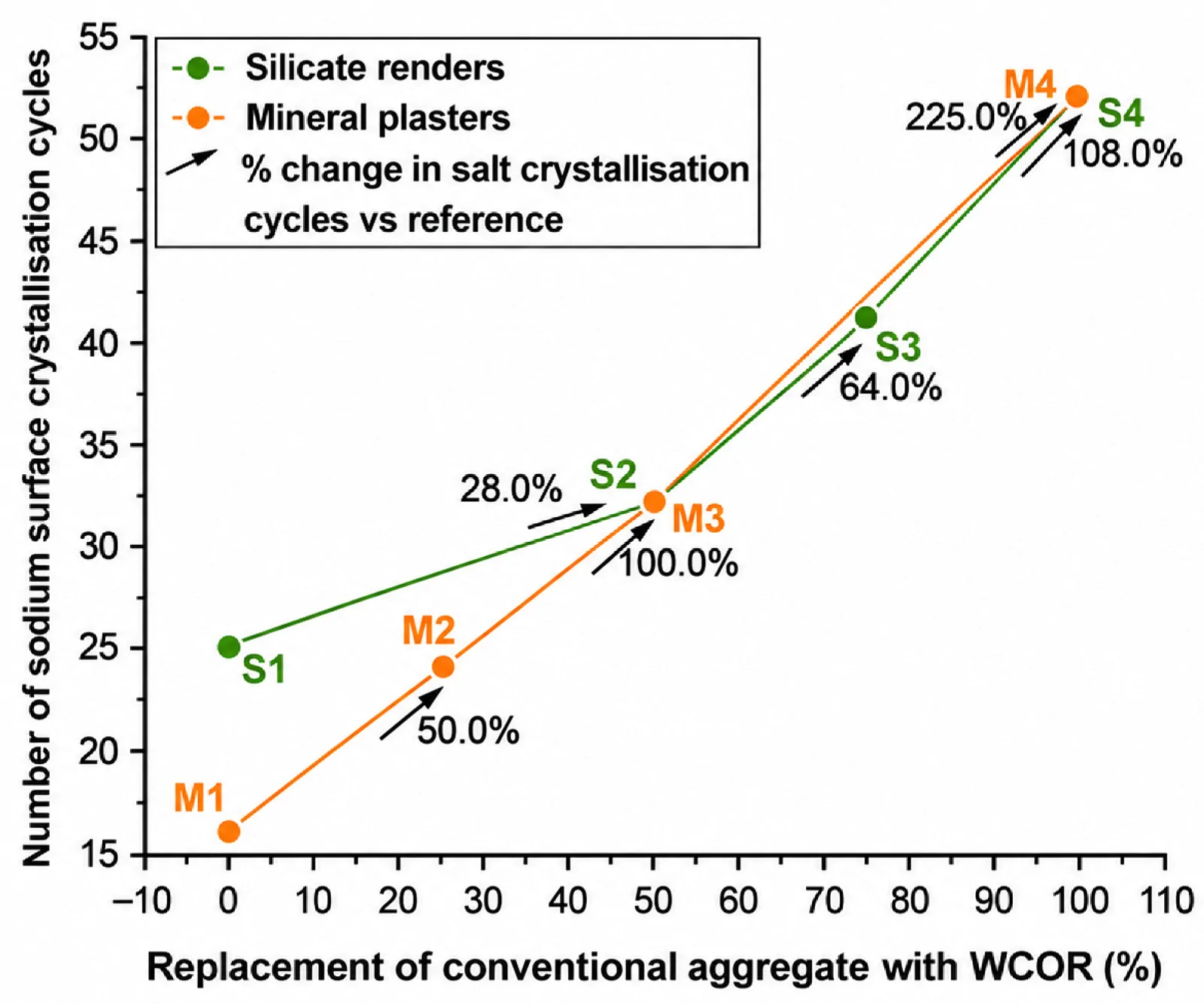
Percentage increase in salt crystallisation cycles relative to the reference renders.

The results of the salt-crystallisation resistance tests are presented in Figure 15. The renders were exposed to a 14% sodium sulphate decahydrate (Na_2_SO_4_·10H_2_O) solution, and the test was continued until failure occurred. The percentage changes in the number of sodium sulphate crystallisation cycles with increasing aggregate replacement levels, calculated relative to the corresponding reference samples (S1 and M1), are presented in Figure 16.

Increasing the WCOR content improved resistance to sodium sulphate crystallisation in both render types. The number of cycles increased from 25 for S1 to 52 for S4 in the silicate series and from 16 for M1 to 52 for M4 in the mineral series. However, the lowest mass loss among the silicate renders was obtained for S3, whereas complete replacement in S4 increased the mass loss to 4.5%. In the mineral renders, mass losses remained relatively similar despite the higher number of cycles sustained. The improved resistance may be associated with changes in pore-size distribution, connectivity, and the volume of pores available for salt transport and crystallisation [56,57]. Porous WCOR particles may provide additional space for salt accumulation and reduce local crystallisation stresses. In silicate renders, the polymer dispersion and polysiloxane additive may further limit pore wetting and salt-solution ingress [58,59].

The most favourable balance was obtained for S3, which withstood 41 crystallisation cycles while exhibiting the lowest mass loss of only 1%. Although S4 survived more cycles, its higher mass loss suggested that complete WCOR replacement not only increased salt-accommodation capacity but also promoted more intensive scaling after a critical salt concentration was reached. In the mineral renders, the relatively stable mass loss indicates that WCOR mainly delayed the onset of damage rather than changing the final degradation mechanism.

**Figure 17:**
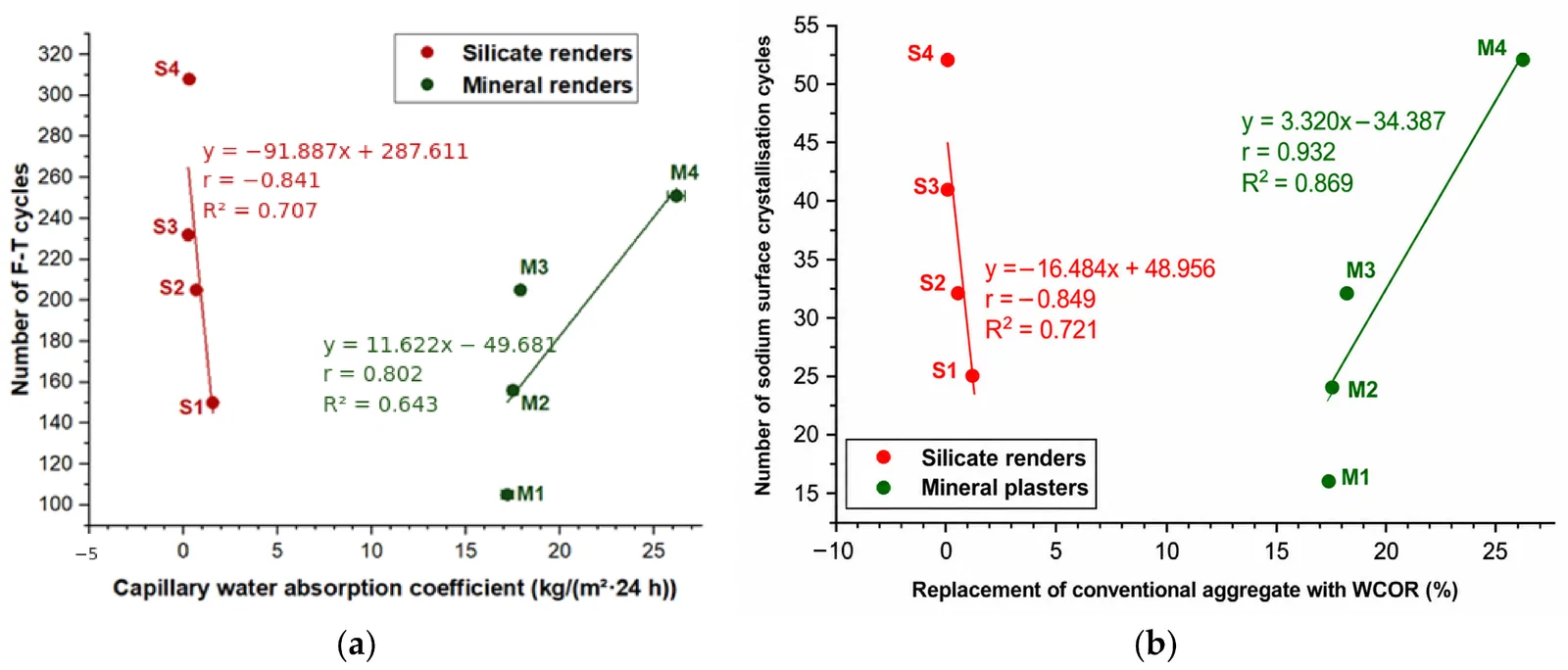
Relationship between the capillary water absorption coefficient and environmental durability of silicate and mineral renders: (**a**) number of freeze–thaw cycles and (**b**) number of sodium sulphate crystallisation cycles. Linear regression was performed separately for the silicate and mineral-render series.

To determine whether the changes in the transport properties of the renders were related to their environmental resistance, the relationships between the capillary water absorption coefficient and both the number of freeze–thaw cycles and the number of sodium sulphate crystallisation cycles were analysed (Figure 17).

For the silicate renders, a negative relationship was observed between the capillary water absorption coefficient and the number of freeze–thaw cycles. As capillary water absorption decreased, the number of F–T cycles sustained by the renders increased. A similar relationship was found for resistance to sodium sulphate crystallisation. This indicates that, in the silicate renders, reduced liquid-water transport was accompanied by improved performance with respect to both durability indicators considered. The observed trend is consistent with the freeze–thaw degradation mechanism of capillary-porous materials. Water penetrating the pore structure increases the degree of saturation of the material, while subsequent freezing may generate hydraulic stresses and promote damage development. However, the literature indicates that the relationship between capillary water uptake and freeze–thaw resistance is not governed solely by the total amount of absorbed water. Pore size, pore-size distribution, pore continuity, and the ability of water to migrate towards available voids during freezing are also of major importance.

An opposite relationship was obtained for the mineral renders. The capillary water absorption coefficient showed a positive correlation with the number of F–T cycles. A positive relationship was also observed between capillary water absorption and resistance to salt crystallisation. Thus, within the mineral-render group, a greater capacity for liquid-water transport was accompanied by a greater number of degradation cycles sustained.

In the mineral renders, freeze–thaw and salt-crystallisation resistance increased despite higher capillary water absorption. This shows that water absorption alone did not determine their durability. Previous studies have shown that damage caused by freezing and salt crystallisation also depends on pore-size distribution, pore connectivity, and the degree of water saturation [53,56,60]. However, these properties were not measured in the present study and require further investigation.

#### 3.2.5. Adhesion to the Substrate and Impact Resistance

The results of the render–substrate adhesion tests and impact resistance tests are presented in Table 5.

The mean adhesion strength of the silicate renders varied only slightly, from 0.27 N/mm^2^ for S1 to 0.30 N/mm^2^ for S4. Because the error ranges overlapped, this change was treated as a descriptive trend rather than evidence of a statistically significant difference among the formulations. All silicate renders maintained their integrity during the impact-resistance test, with no visible damage or debonding.

An opposite and more pronounced trend was observed for the mineral renders, whose mean adhesion strength decreased from 0.31 N/mm^2^ for M1 to 0.19 N/mm^2^ for M4. This decrease was accompanied by local damage and debonding of the render layer after impact. The different responses of the two render families may be associated with differences in their binder composition, including the presence of polymer dispersion in the silicate renders. However, the contribution of individual components and changes in the render–substrate interfacial zone were not investigated separately.

The favourable adhesion of the silicate renders may be attributed to styrene–acrylic dispersion, which can improve the render–substrate interfacial zone, reduce shrinkage, and reinforce the bond through polymer-film formation [61,62,63]. In the mineral renders, lower adhesion may have resulted from reduced flowability and poorer wetting of substrate irregularities. However, pull-off results should be interpreted together with the failure mode, as the measured strength may reflect either interfacial adhesion or cohesion within the render layer. The absence of impact damage in the silicate renders, compared with cracking and debonding in the mineral renders, also suggests that dynamic resistance depended on matrix deformability and the crack-arrest capacity. The polymer phase may have improved fracture resistance and energy dissipation under impact loading [62]. Polymer dispersions can improve mortar adhesion, cohesion, and crack resistance by forming a polymeric phase that strengthens the interfacial region [61,64,65]. Similar improvements in interlayer adhesion have been reported for renders containing EVA copolymer and porous waste materials [66]. Styrene–polyacrylic latex has also been shown to improve workability and promote a denser, better-integrated microstructure [64]. In the silicate renders, the styrene–acrylic dispersion may therefore have partly compensated for the reduced flowability associated with WCOR, while the rough opoka particles may have additionally enhanced mechanical interlocking.

In the mineral renders, reduced flowability was accompanied by lower adhesion, probably due to poorer wetting and the penetration of substrate irregularities. Water retention by porous WCOR particles may have further reduced the amount of free water available at the render–substrate interface [65].

**Figure 18:**
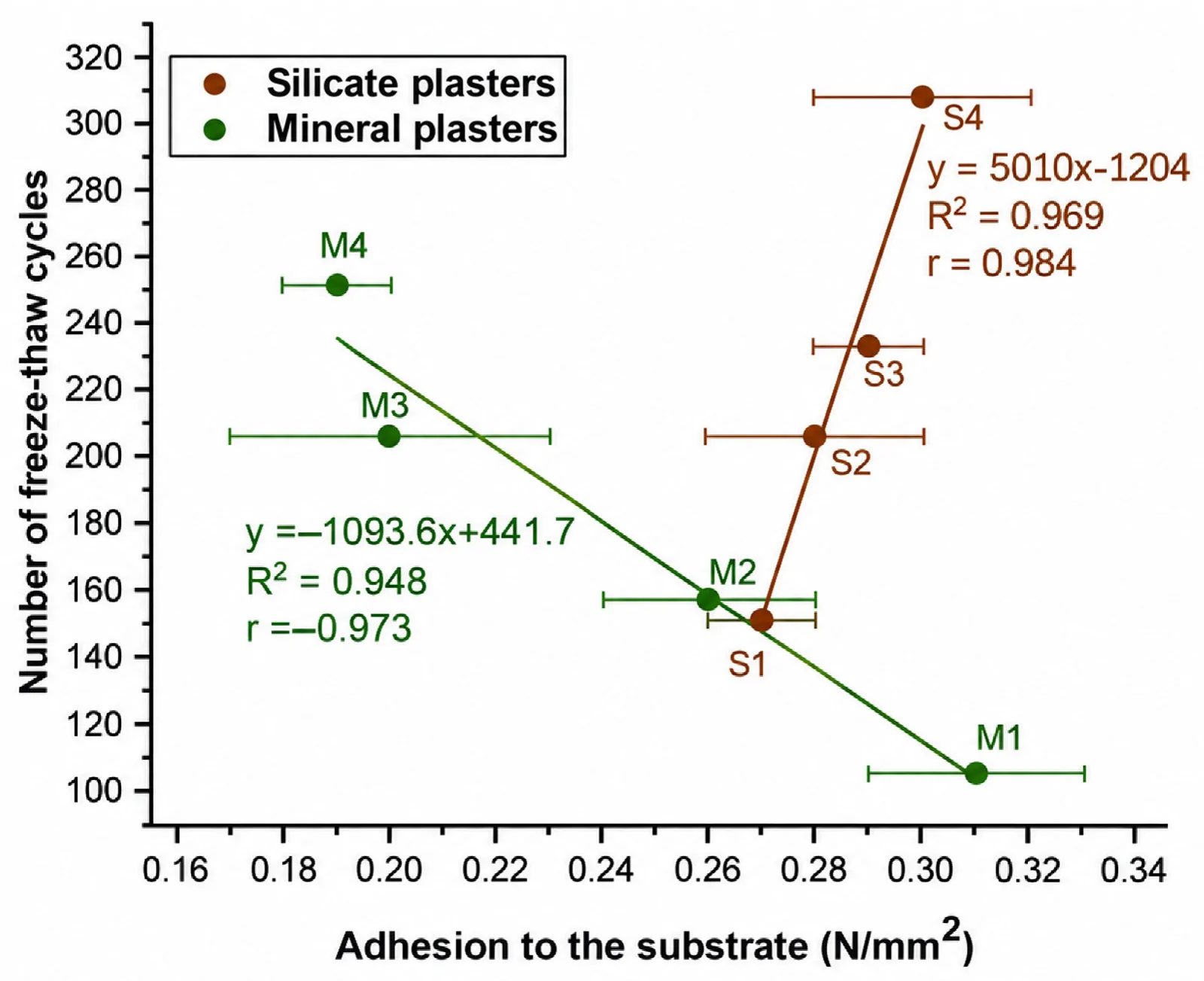
Exploratory formulation-level relationships between mean adhesion strength and the number of freeze–thaw cycles for silicate and mineral renders.

Exploratory formulation-level relationships between the mean adhesion strength and freeze–thaw resistance were examined separately for the two render families (Figure 18). Because each relationship was based on only four formulation means (*n* = 4), Pearson correlation coefficients were used solely as descriptive measures of the observed trends and not as inferential evidence.

For the silicate renders, formulations with higher mean adhesion strength also sustained a greater number of freeze–thaw cycles, resulting in a positive Pearson correlation coefficient (r = 0.984). This trend is consistent with the possibility that preservation of the render–substrate interfacial zone and the integrity of the render layer contributed to improved resistance under cyclic freezing and thawing. Previous studies have shown that the freeze–thaw degradation of mortars may involve microcracking within the matrix, weakening of the interfacial zone, delamination, and debonding of the render layer [67,68]. However, because the relationship was based on only four formulations, the present results did not confirm this mechanism.

An opposite trend was observed for the mineral renders. In this group, formulations with a lower mean adhesion strength sustained a greater number of freeze–thaw cycles, resulting in a negative Pearson correlation coefficient (r = −0.973). This observation indicates that the improvement in freeze–thaw resistance was not accompanied by improved adhesion to the concrete substrate. Other material characteristics, potentially including pore-size distribution, pore connectivity, the degree of water saturation, and moisture-transport behaviour, may have contributed to the observed durability response [68]. However, these characteristics were not quantified in the present study and should therefore be regarded as possible explanatory factors rather than demonstrated mechanisms.

Overall, the two render families exhibited opposite formulation-level trends. A higher mean adhesion was accompanied by greater freeze–thaw resistance in the silicate renders, whereas the freeze–thaw resistance of the mineral renders increased despite decreasing adhesion. Because both correlations are based on only four formulation means, these relationships should be treated as exploratory and require confirmation using a larger independent set of formulations.

#### 3.2.6. UV Ageing Test

Table 6 presents the initial values of capillary water absorption and adhesion strength, together with the corresponding ranges of change after accelerated UV exposure and wind-driven rain.

The silicate and mineral renders clearly exhibited different responses to environmental ageing. In the silicate series, deterioration in both water-transport properties and adhesion are relatively limited, which may be attributed to the initially low capillary absorption and the protective effect of polymeric and hydrophobic constituents. S3, containing 75% WCOR, showed the most stable behaviour, combining the lowest initial capillary water absorption (0.25 kg/(m^2^·24 h)) with relatively high adhesion strength (0.29 N/mm^2^) and only limited deterioration after ageing.

Although S4 exhibited the highest freeze–thaw resistance, its stability under prolonged cyclic wetting, drying, and UV exposure was lower than that of S3. Complete replacement with porous WCOR may have increased moisture retention and modify transport pathways, resulting in a greater increase in water absorption during ageing. This confirms that maximum freeze–thaw resistance does not necessarily correspond to optimum resistance under other environmental exposures. Similar composition-dependent changes in water absorption, surface properties, and the microstructure of thin-layer renders during ageing have been reported previously [18,68].

The mineral renders showed substantially higher initial capillary absorption and a progressive decrease in adhesion with increasing WCOR content. M4 exhibited the least favourable combination, with a capillary absorption of 26.2 kg/(m^2^·24 h) and an adhesion strength of only 0.19 N/mm^2^, resulting in greater susceptibility to degradation during repeated wetting, drying, and surface heating [19]. In contrast, M2 provided a better balance between adhesion and the previously observed improvement in freeze–thaw and salt-crystallisation resistance.

Overall, S3 can be considered the balanced-performance optimum, combining low water absorption, good adhesion, and high stability after environmental ageing, whereas S4 represents a durability-driven optimum, primarily in terms of freeze–thaw resistance. For the mineral renders, the results similarly indicate that complete WCOR replacement should not automatically be regarded as optimal for long-term façade performance, as the improvement in freeze–thaw resistance is accompanied by poorer moisture-related performance and adhesion.

#### 3.2.7. Microstructure—Scanning Electron Microscopy

**Figure 19:**
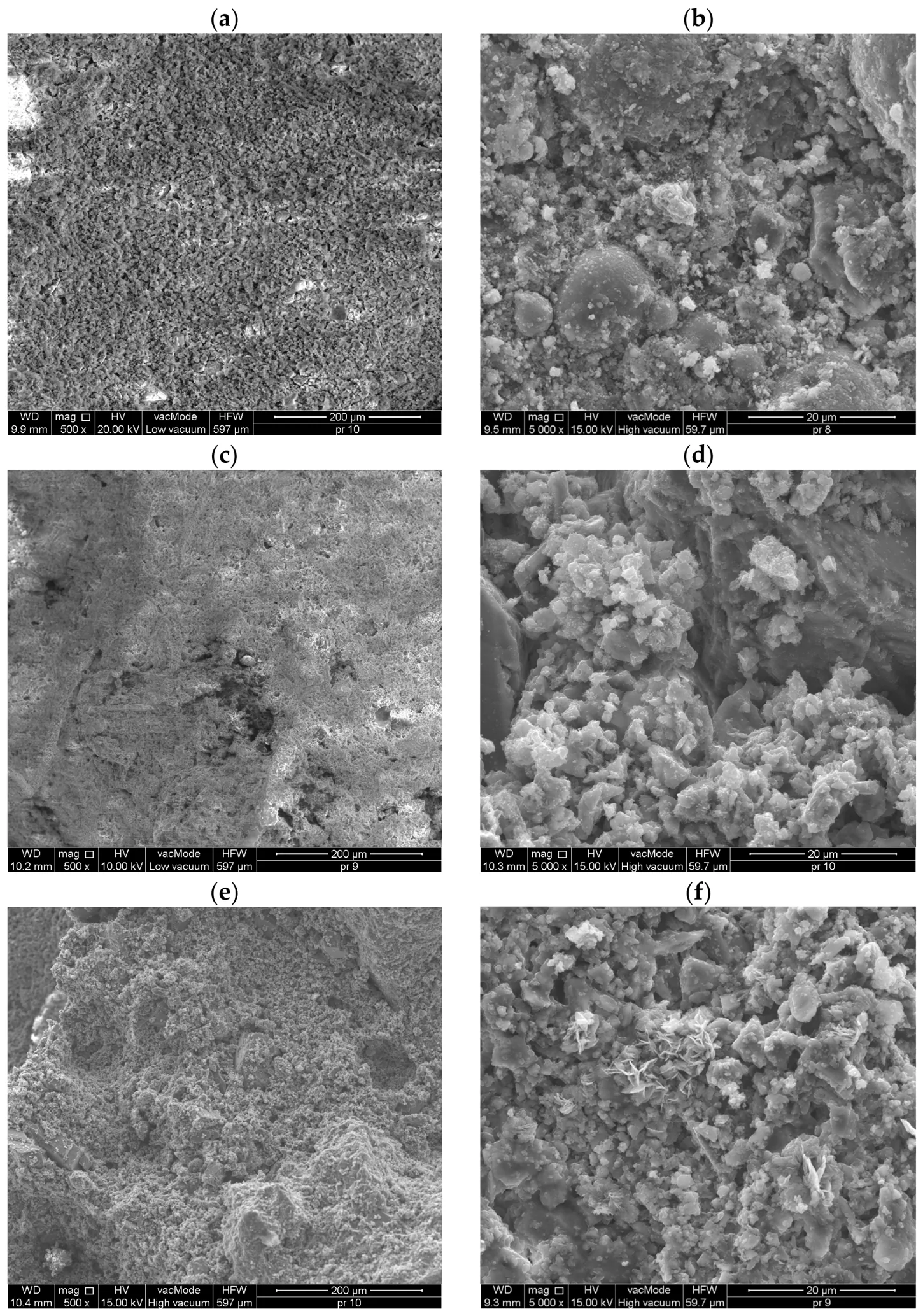
Microstructures of the reference and WCOR-modified silicate renders: (**a**) S1—500×; (**b**) S1—5000×; (**c**) S3—500×; (**d**) S3—5000×; (**e**) S4—500×; (**f**) S4—5000×.

**Figure 20:**
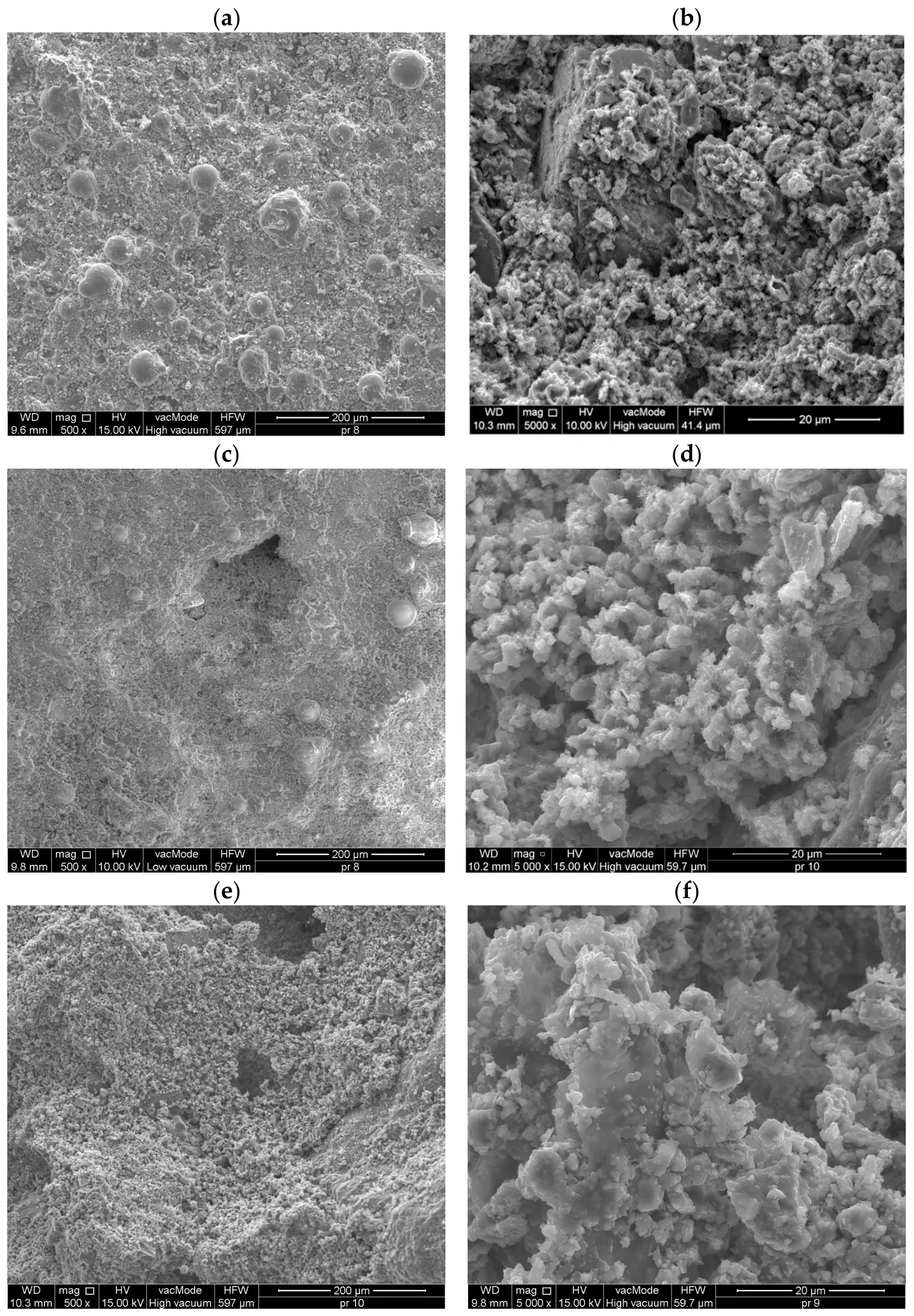
Microstructures of the reference and WCOR-modified mineral renders: (**a**) M1—500×; (**b**) M1—5000×; (**c**) M3—500×; (**d**) M3—5000×; (**e**) M4—500×; (**f**) M4—5000×.

The morphology and porous structure of the renders were investigated using scanning electron microscopy (SEM). For SEM examination, fracture fragments of the specimens were mounted on carbon holders using a carbon-based adhesive. The specimens were subsequently coated with an approximately 50 nm thick carbon layer to provide adequate electrical conductivity at the specimen surface. Figure 19 presents the microstructure of selected silicate renders, whereas Figure 20 shows the microstructure of selected mineral renders.

SEM observations combined with EDS microanalysis indicated that the analysed areas contained phases rich in Ca, Al, and Si. In the mineral renders, the elemental compositions and morphologies of some hydration products were consistent with C–S–H, the principal hydration product of cementitious binders. These products occurred predominantly as irregular, approximately isometric, or flattened particles forming relatively compact agglomerates. Their appearance was consistent with the Type III and Type IV C–S–H morphologies described in Diamond’s classification (Figure 20). A well-developed fibrous morphology corresponding to Type II C–S–H was not observed in the analysed SEM images. Because identification was based on particle morphology and local EDS spectra, it should be regarded as indicative rather than definitive.

In the analysed areas of the SEM images, close contact was observed locally between the binder and both the fine sand and WCOR particles. By comparison, local discontinuities were visible at some interfaces between the binder and the Omyadol dolomite particles (Figure 20d). The exposed Omyadol particles had relatively smooth fracture surfaces, which may have provided less favourable conditions for mechanical interlocking than the irregular and more developed surfaces of WCOR particles. However, these observations were qualitative and did not provide a quantitative assessment of interfacial bond quality.

At 500× magnification, the WCOR-containing renders showed more visible voids, irregular interparticle spaces, and more heterogeneous fracture surfaces than the corresponding reference renders (Figure 19a,c,e and Figure 20a,c,e). Images obtained at 5000× magnification provided further details of the local matrix morphology and aggregate–binder contact. However, because the micrographs represent select two-dimensional areas of the fracture surfaces, they cannot be used to quantify total porosity, pore-size distribution, or pore connectivity.

The reference silicate render S1 exhibited a comparatively homogeneous matrix in the analysed SEM images, with fewer clearly visible voids and discontinuities. The fracture surface of S3 was more heterogeneous and contained more regions with an irregular granular morphology. These features were also visible in S4, in which larger agglomerates, irregular interparticle spaces, and a more developed fracture-surface topography were observed. The SEM images therefore indicate local morphological differences associated with increasing WCOR content. They do not, however, demonstrate systematic changes in the three-dimensional pore structure.

A somewhat different local morphology was observed in the mineral renders. The reference render M1 exhibited a comparatively continuous matrix with locally distributed voids. The fracture surfaces of M3 and M4 showed more irregular agglomerates and visible spaces between hydration products and aggregate particles. Among the examined mineral renders, M4 had the most heterogeneous fracture-surface appearance. These qualitative observations suggest that the incorporation of WCOR affected the local morphology of the mineral-render matrix. However, they do not demonstrate changes in total porosity or enhanced water redistribution during freeze–thaw and salt-crystallisation exposure. A more detailed assessment of its effects on pore structure, moisture redistribution, and cyclic durability requires additional quantitative investigations, such as MIP, micro-CT, or quantitative image analysis.

### 3.3. Integrated Performance Assessment

The individual test results revealed that increasing WCOR content produced both beneficial and adverse changes. Therefore, the formulations were additionally assessed using a multi-parameter performance map, multi-criteria ranking, weighting sensitivity analysis, and Pareto analysis. These complementary approaches were used to distinguish formulations maximising cyclic durability from those providing a more balanced combination of moisture transport, adhesion, durability, and application-related properties.

#### 3.3.1. Multi-Parameter Performance Map

**Figure 21:**
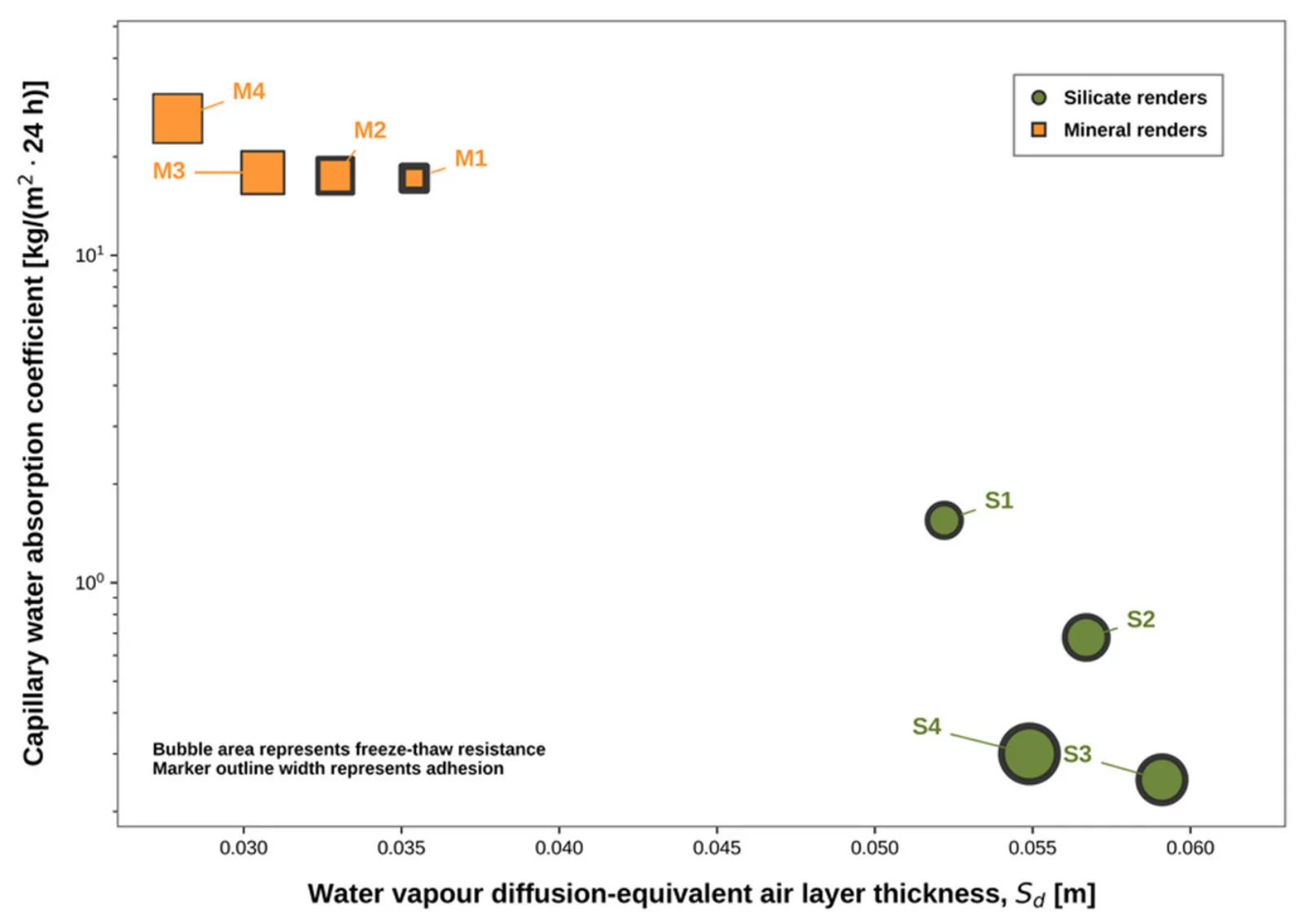
Multi-parameter moisture transport–durability map of silicate and mineral renders containing waste calcareous opoka rock (WCOR). The *x*-axis represents the water-vapour diffusion-equivalent air-layer thickness (S_d_), while the *y*-axis shows the capillary water absorption coefficient on a logarithmic scale. Marker area is proportional to freeze–thaw resistance, expressed as the number of cycles to first visible damage, whereas marker outline width represents adhesion to the substrate. Circles denote silicate renders and squares denote mineral renders.

Figure 21 presents a multi-parameter performance map illustrating the relationships among moisture transport, freeze–thaw durability, and render adhesion. The *X*-axis shows the water-vapour diffusion-equivalent air-layer thickness, Sd, while the *Y*-axis represents the capillary water absorption coefficient on a logarithmic scale. Marker size corresponds to the number of freeze–thaw cycles sustained, and border thickness indicates adhesion strength. Circles denote silicate renders and squares denote mineral renders.

The plot clearly separates the two render families, confirming that the effect of WCOR depended strongly on the matrix type. Silicate renders were characterised by very low capillary water absorption and higher Sd values, whereas mineral renders showed much greater liquid-water uptake and lower resistance to water-vapour diffusion.

In both groups, increasing WCOR content increased freeze–thaw resistance, as reflected by the larger marker size. In the silicate series, this improvement was accompanied by maintained low capillary absorption and slightly improved adhesion, indicating a favourable combination of durability, limited liquid-water transport, and good interfacial integrity.

A different response was observed for the mineral renders. Despite the increase in capillary water absorption and the gradual reduction in adhesion strength with increasing WCOR content, freeze–thaw resistance improved markedly. This behaviour is particularly evident for M4, which is represented by the largest marker but a relatively thin outline. This result indicates that the higher freeze–thaw resistance of the mineral renders was not accompanied by either lower capillary water absorption or higher adhesion strength. A complete explanation of this response requires additional quantitative characterisation of the pore structure and moisture distribution.

Overall, the map shows that no single measured parameter was sufficient to describe the durability response of all WCOR-containing renders. The relationships among moisture transport, adhesion, and freeze–thaw resistance differed between the silicate and mineral matrices.

#### 3.3.2. Multi-Criteria Performance Ranking

The influence of WCOR on render performance was multidirectional and strongly dependent on the matrix type. Increasing WCOR content improved freeze–thaw and sodium sulphate crystallisation resistance in both render families, whereas changes in moisture transport and adhesion were less uniform.

The trade-off was most evident at the highest replacement level. S4 showed the highest freeze–thaw resistance, while S3 provided lower capillary water absorption and the lowest salt-crystallisation mass loss. Similarly, M4 maximised freeze–thaw resistance but simultaneously exhibited the highest capillary water absorption and the lowest adhesion strength.

These results show that increasing WCOR content does not optimise all properties simultaneously. Therefore, a multi-criteria assessment was applied to distinguish between a durability-oriented optimum, focused on resistance to degradation, and a balanced-performance optimum, reflecting the best overall combination of transport, durability, adhesion, and application-related properties.

Multi-criteria ranking.

The PI values and their corresponding interpretation are summarised in Table 7. For both render types, the highest PI was obtained at 100% replacement of conventional aggregate with WCOR. This result does not, however, imply that the S4 and M4 formulations are unconditionally the most suitable for practical application because both exhibit the highest class of resistance during manual texturing (AR = 3).

**Figure 22:**
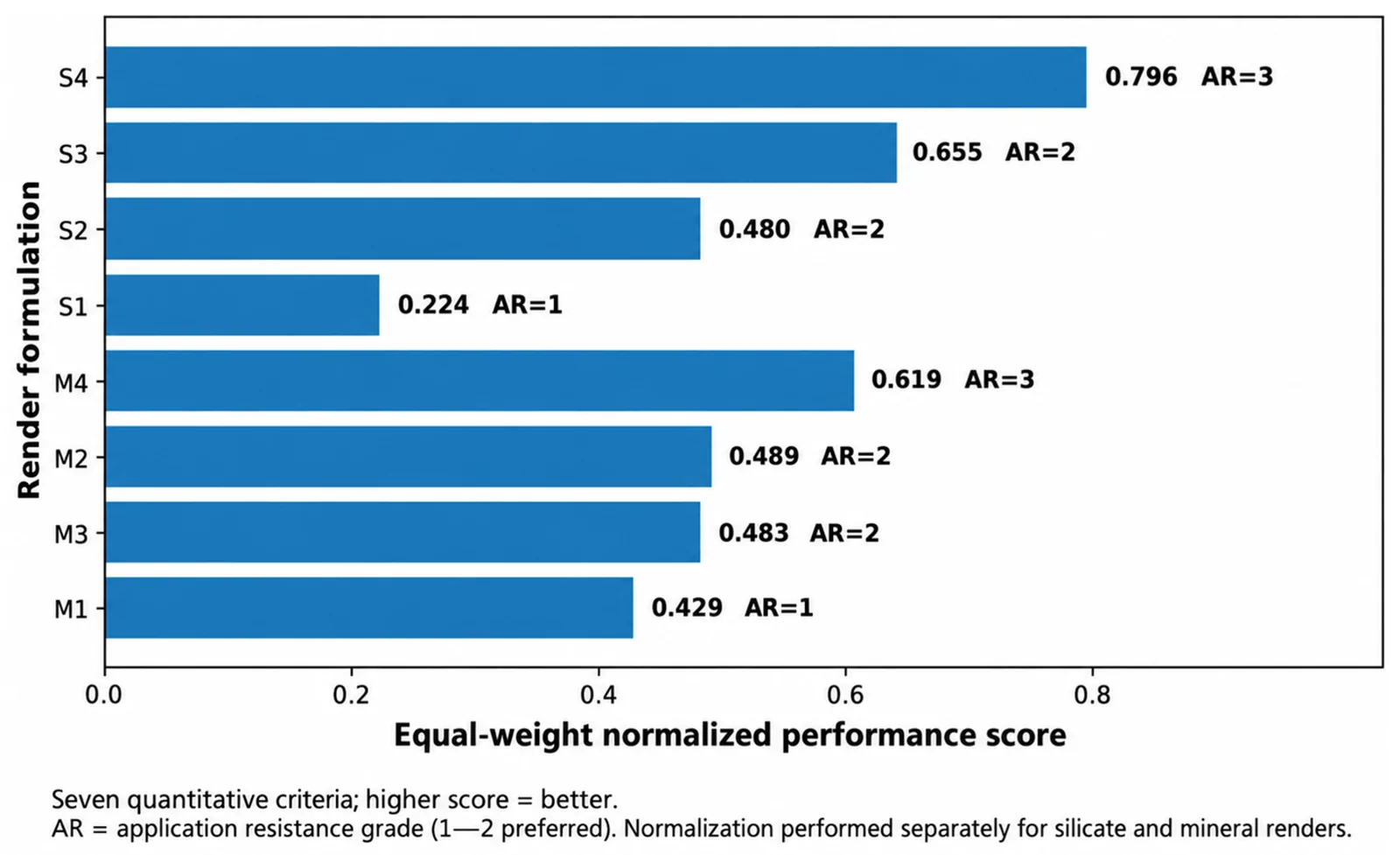
Multi-criteria performance ranking within each render matrix. Seven quantitative criteria were normalised on a 0–1 scale and combined using equal weights; AR denotes the application resistance grade.

Within the silicate-render series, the following ranking was obtained: S4 > S3 > S2 > S1 (Figure 22).

The PI increased from 0.224 for S1 to 0.796 for S4. The S4 formulation, containing 100% WCOR, showed the highest cyclic durability, withstanding 308 freeze–thaw cycles and 52 sodium sulphate crystallisation cycles, while maintaining high adhesion strength (0.30 N/mm^2^). However, its salt-crystallisation mass loss increased to 4.5% and application resistance reached AR = 3. Therefore, S4 should be regarded primarily as a durability-driven optimum rather than the best overall formulation. S3 achieved the second-highest PI (0.655) and provided a more balanced performance profile. It combined the lowest capillary water absorption (0.25 kg/(m^2^·24 h)) and salt-crystallisation mass loss (1.0%) with an adhesion strength close to that of S4 (0.29 N/mm^2^) and lower application resistance (AR = 2). Thus, S3 represents the balanced-performance optimum, whereas S4 maximises resistance to cyclic degradation.

**Figure 23:**
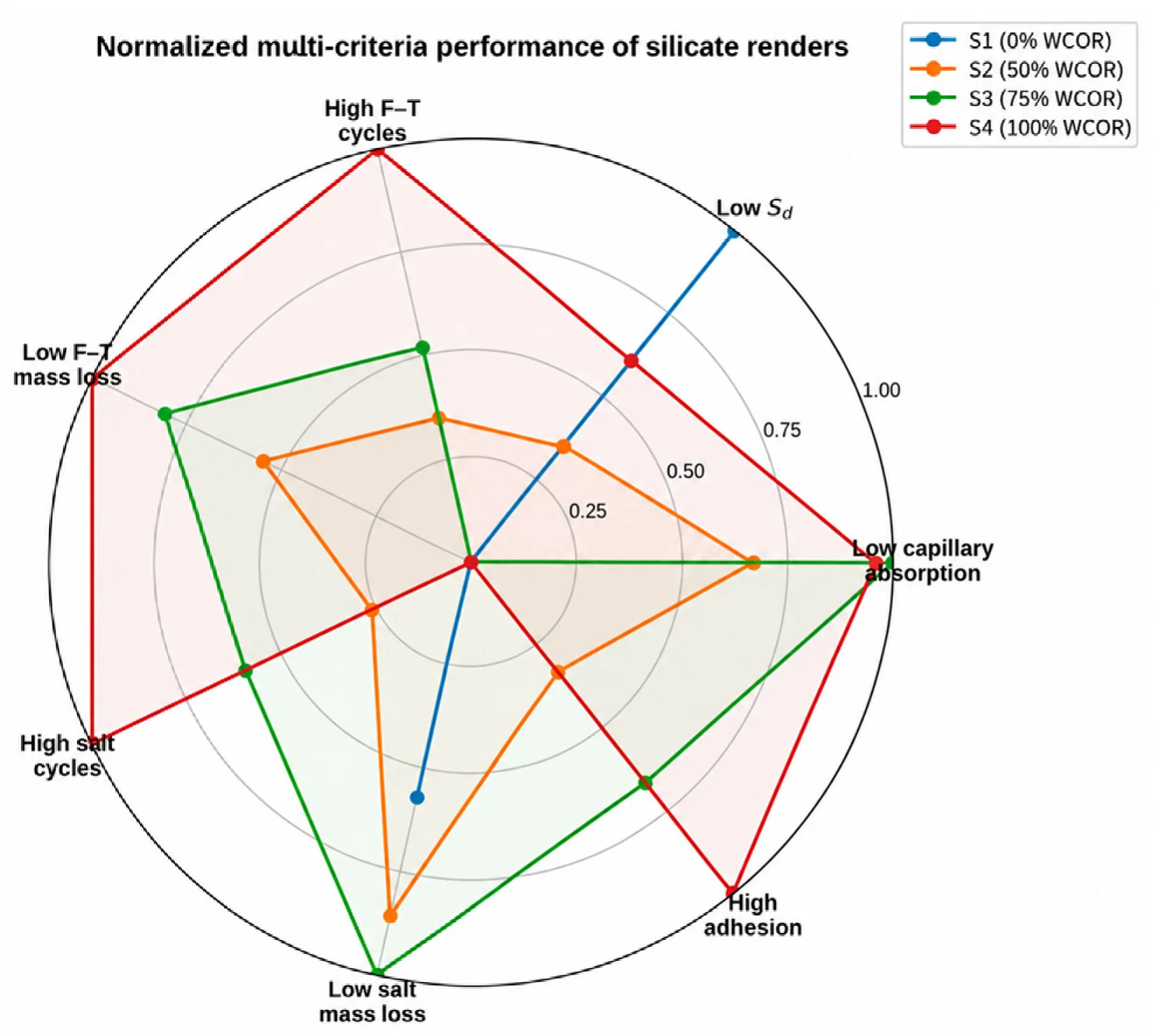
Normalised multi-criteria performance of silicate renders with different WCOR replacement levels. A value of 1 denotes the most favourable result within the silicate-render series for a given criterion.

The radar chart (Figure 23) confirms this trade-off, showing that increasing WCOR replacement from 75% to 100% further improves cyclic durability but does not provide corresponding improvements in moisture-related and mass-stability parameters.

**Figure 24:**
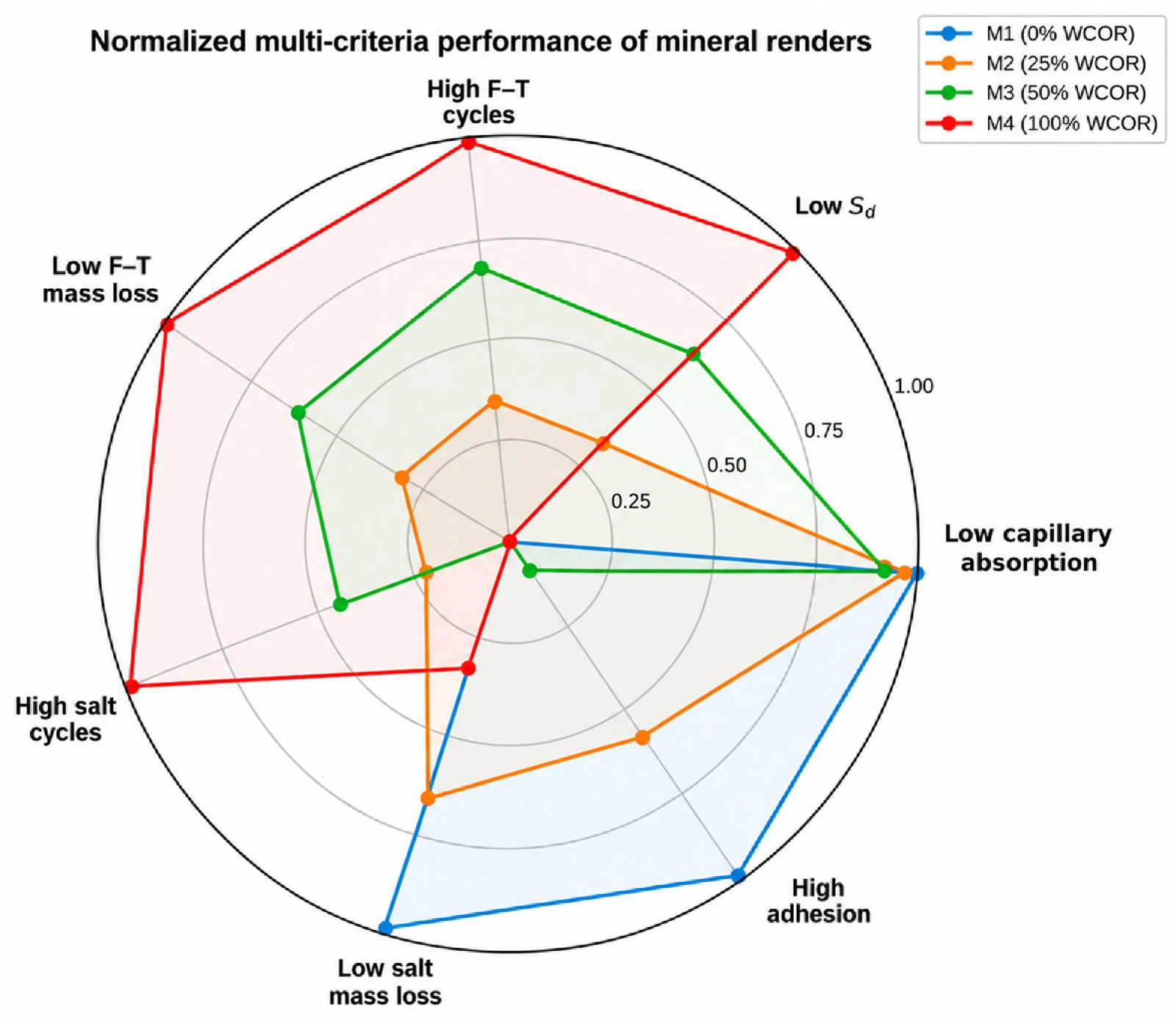
Normalised multi-criteria performance of mineral renders with different WCOR replacement levels. A value of 1 denotes the most favourable result within the mineral-render series for a given criterion.

Within the mineral-render series (Figure 24), M4 achieved the highest PI (0.619), mainly due to its superior freeze–thaw and salt-crystallisation resistance and the lowest Sd value. However, it also showed the highest capillary water absorption (26.2 kg/(m^2^·24 h)), the lowest adhesion strength (0.19 N/mm^2^), and the highest application resistance. Thus, complete WCOR replacement improved environmental durability but impaired selected service-related properties.

The PI values of M2 and M3 were very similar (0.489 and 0.483), indicating no clear overall advantage of either formulation. M2 provided better adhesion and slightly lower water absorption, whereas M3 showed higher resistance to cyclic environmental degradation. Therefore, the 25–50% WCOR replacement range represents a practical compromise, while M4 is preferable only when durability and vapour permeability are prioritised over adhesion, water uptake, and application performance.

The M2 and M3 formulations occupy intermediate positions, confirming that, for the mineral renders, there is no single WCOR replacement level that simultaneously maximises all required performance characteristics.

#### 3.3.3. Sensitivity Analysis to the Adopted Priorities

Because the outcome of a multi-criteria assessment may depend on the relative importance assigned to individual criteria, the stability of the ranking was evaluated using three weighting scenarios. The first scenario assigned equal weights to all seven criteria (w_j_ = 1/7 = 0.143). In the durability-oriented scenario, the four criteria associated with cyclic deterioration, i.e., freeze–thaw resistance, freeze–thaw mass loss, salt-crystallisation resistance, and salt-crystallisation mass loss, were assigned twice the weight of capillary water absorption, Sd, and adhesion. After normalisation, the corresponding weights were 0.182 and 0.091, respectively. In the moisture-transport-and-adhesion-oriented scenario, capillary water absorption, Sd, and adhesion were assigned twice the weight of the four durability criteria, resulting in weights of 0.200 and 0.100, respectively.

For each formulation, the weighted performance index was calculated as:

(10) $$PI_{i}=\;\sum_{j=1}^{n}w_{j}N_{ij},\;\;\;\;\;\;\sum_{j=1}^{n}w_{j}=1,\;\;\;\;\;\;\;n=7\;\;$$

where *N_ij_* denoted the normalised performance of formulation *i* with respect to criterion j and *Σw_j_ =* 1. The analysis was performed both without and with the practical application constraint AR ≤ 2. This constraint was treated as a feasibility condition rather than an additional performance criterion, because AR described the resistance encountered during manual texturing and therefore determined whether the formulation remained practically applicable. The weighting scenarios used in the sensitivity analysis are shown in Table 8.

Within the silicate-render series, S4 remained the highest-ranked formulation under all three weighting scenarios when no application-related constraint was imposed, confirming its superior durability-oriented performance. However, S4 also exhibited the highest manual-texturing resistance (AR = 3). After imposing the practical condition AR ≤ 2, S3 was consistently ranked first irrespective of the adopted weighting scenario. Thus, S3, containing 75% WCOR, can be regarded as a robust, balanced-performance optimum rather than an optimum resulting specifically from the equal-weight assumption. S4 should instead be interpreted as the durability-driven optimum, for which improved resistance to cyclic degradation is accompanied by increased application resistance.

A different response was observed for the mineral renders. M4 remained the highest-ranked formulation when application resistance was not considered. After introducing the AR ≤ 2 constraint, however, the selected formulation became dependent on the engineering priorities. M2 was ranked highest under equal weighting and under the moisture/adhesion-oriented scenario, whereas M3 became preferable under durability-oriented weighting. Consequently, the difference between M2 and M3 should not be interpreted as evidence of a unique optimum. Instead, the 25–50% WCOR replacement interval represents a robust, balanced-performance zone, within which M2 is preferable when adhesion and moisture-related properties are prioritised, whereas M3 is preferable when cyclic durability is of greater importance. The sensitivity of the selected optimum to criterion weighting and the practical application constraint AR ≤ 2 is shown in Table 9.

The sensitivity analysis therefore shows that the main interpretation is stable for the silicate renders once practical application is taken into account, whereas the mineral-render series exhibits a genuine priority-dependent trade-off. Accordingly, the revised interpretation distinguishes between a durability-driven optimum and a balanced-performance optimum and avoids presenting the equal-weight ranking as the only basis for formulation selection.

#### 3.3.4. Pareto Analysis and the Nature of the Trade-Offs

The multi-criteria ranking was further complemented by a Pareto analysis. In the full seven-criterion space, none of the formulations within a given matrix dominated all the others simultaneously because each formulation retained an advantage in at least one performance parameter. This is an important result because it mathematically confirms the existence of a genuine trade-off between durability, moisture transport, and adhesion, rather than a simple relationship of the “more WCOR = better” type.

**Figure 25:**
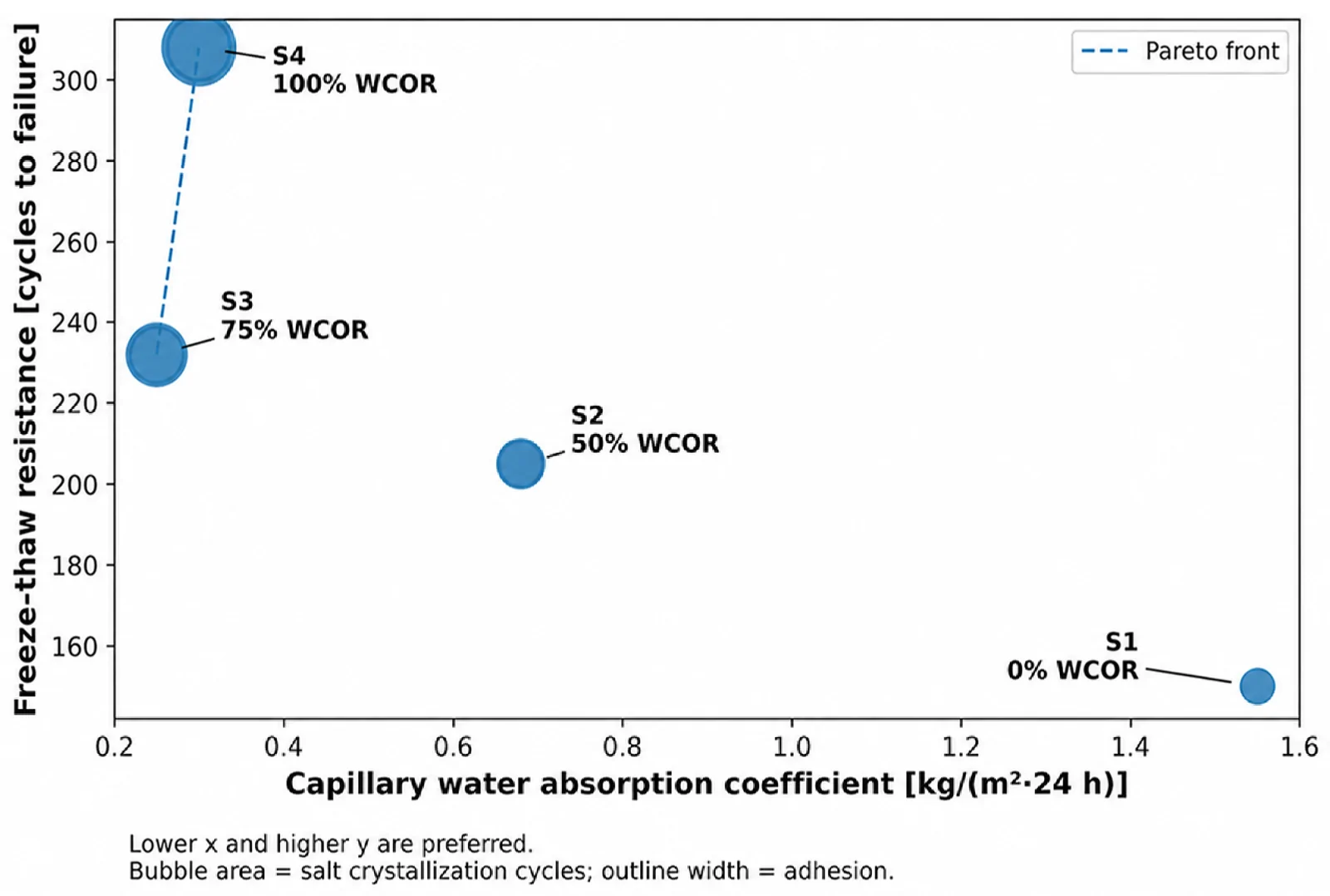
Reduced Pareto view for silicate renders: capillary water absorption versus freeze–thaw resistance. Lower water absorption and higher freeze–thaw resistance are preferred; the bubble area represents salt crystallisation cycles and marker outline width represents adhesion.

**Figure 26:**
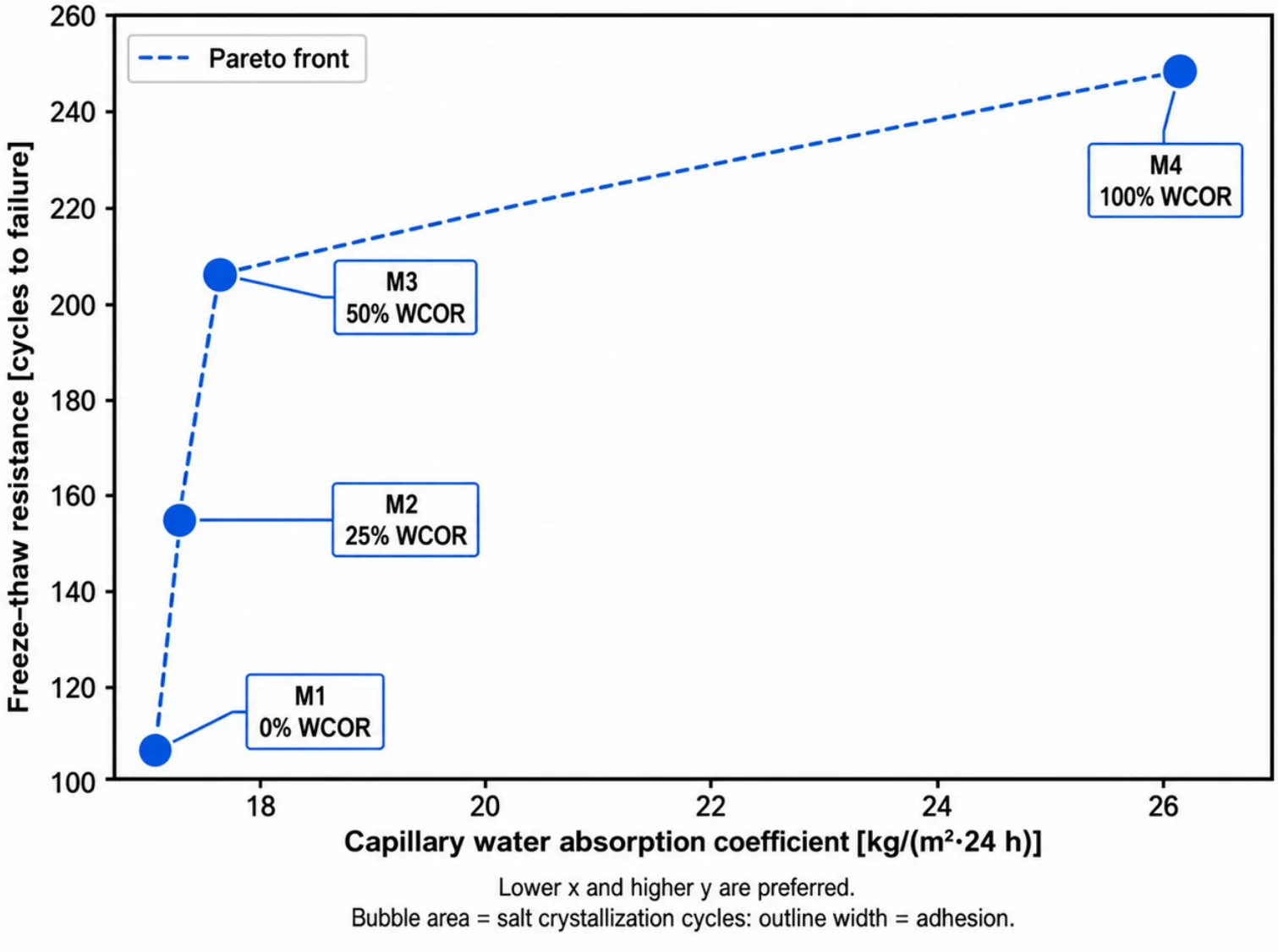
Reduced Pareto view for mineral renders: capillary water absorption versus freeze–thaw resistance. Lower water absorption and higher freeze–thaw resistance are preferred; the bubble area represents salt crystallisation cycles and marker outline width represents adhesion.

For clearer visualisation, an additional two-dimensional Pareto projection was prepared, in which capillary water absorption was minimised and freeze–thaw resistance was maximised (Figure 25 and Figure 26). The marker size represented the number of salt-crystallisation cycles, whereas the border thickness represented adhesion strength. This projection does not replace the full seven-criterion analysis but provides a clear visual representation of the fundamental conflict between water uptake and freeze–thaw resistance.

Within the silicate-render series, S1 and S2 are dominated by S3 in this projection, whereas the Pareto front is formed by S3 and S4. S3 represents the solution oriented towards minimising liquid-water transport, while S4 represents the formulation that maximises resistance to freeze–thaw cycling. The comparison between these two formulations clearly illustrates the distinction between the balanced-performance optimum and the durability-driven optimum.

For the mineral renders, all formulations M1–M4 remained on the two-dimensional Pareto front because each increase in freeze–thaw resistance was accompanied by an increase in capillary water absorption. This indicates a systematic trade-off between these two properties: the improvement in freeze–thaw resistance was not accompanied by reduced water uptake. This behaviour may be related to pore-structure characteristics and moisture redistribution during freezing; however, these variables were not measured in the present study and require further quantitative investigation.

Overall, the results distinguish between durability-oriented and balanced-performance options. Complete replacement with WCOR maximised the resistance to cyclic freeze–thaw and salt exposure within both investigated render series but was accompanied by the highest manual-texturing resistance and, in the mineral renders, by increased capillary water absorption and reduced adhesion. After applying the AR ≤ 2 constraint, S3 containing 75% WCOR was the most stable balanced-performance option for the silicate renders. For the mineral renders, the preferred formulation varied between M2 and M3 depending on the adopted priorities, identifying 25–50% WCOR as the balanced-performance range. These results are specific to the investigated criteria, normalisation procedure, weighting scenarios, and render formulations.

## 4. Conclusions

The following conclusions were drawn from the results obtained under the laboratory conditions adopted in this study:

- Replacing dolomite aggregate with waste calcareous opoka rock (WCOR) changed the fresh-state properties of both render families. Increasing WCOR content reduced bulk density and flow diameter and increased resistance during manual texturing. The replacement level should therefore be selected with consideration of both hardened-render performance and the ease of application.
- The effect of WCOR on capillary water absorption was matrix-dependent. The silicate renders retained low water absorption, with the lowest value obtained at 75% replacement. In contrast, capillary water absorption increased with WCOR content in the mineral renders, showing that the response depended on both the waste aggregate and the binder system.
- The number of freeze–thaw and salt-crystallisation cycles to first visible damage increased with WCOR content in both render families. In the mineral renders, this improvement occurred despite higher capillary water absorption, indicating that water uptake alone did not determine cyclic resistance. Further investigation of pore structure, moisture distribution, and degree of saturation is required to explain this response.
- Qualitative SEM/EDS observations showed matrix-dependent differences in local fracture-surface morphology and aggregate–binder contact. However, pore structure and moisture redistribution were not quantified, and the chemical reactivity of WCOR was not investigated. These aspects require additional phase and pore-structure analyses.
- The multi-criteria assessment showed that complete WCOR replacement did not provide the most balanced performance. Although 100% WCOR produced the greatest cyclic resistance, it was associated with the highest manual-texturing resistance and, in the mineral renders, with higher capillary water absorption and lower adhesion. Under the adopted criteria, weighting scenarios, and application-related constraints, 75% WCOR provided the most balanced performance in the silicate renders. For the mineral renders, the preferred range was 25–50%: 25% was more favourable when adhesion and moisture-related properties were prioritised, whereas 50% was preferred when cyclic resistance received greater weight.
- The study concerned thin-layer renders applied to rigid concrete substrates. The thermal-insulation layer was not modified, complete ETICS assemblies were not tested, and thermal properties were not measured. The results should therefore not be interpreted as evidence of the thermal or durability performance of complete ETICS. Before practical application, the selected formulations should be validated in complete ETICS assemblies.

Overall, WCOR can be considered a potential mass-based replacement for 0.5–1.5 mm granulated dolomite aggregate in thin-layer renders. Within the scope of this study, 75% WCOR was the most balanced option for the silicate renders, whereas 25–50% was the preferred range for the mineral renders.

## Figures and Tables

**Table 1:** Omyadol^®^ 0.5–1 aggregate grain sizes.

Aggregate Fraction, mm	Share in Aggregate, %
0–0.125	26
0.125–0.25	19.8
0.25–0.5	15.1
0.5–1.0	39.1

**Table 2:** Composition of the silicate renders, expressed as mass percentages of the total wet formulation.

Constituents	S1 wt.%	S2 wt.%	S3 wt.%	S4 wt.%
Water	7.36	7.36	7.36	7.36
Acrylic/polycarboxylate dispersing agent	0.12	0.12	0.12	0.12
Methyl cellulose	0.17	0.17	0.17	0.17
Hydrophobic an aqueous emulsion-based defoamer containing alkoxylated compounds and hydrophobic silica	0.21	0.21	0.21	0.21
Titanium dioxide	0.90	0.90	0.90	0.90
Cationic stabilising additive for potassium-silicate-based formulations	0.43	0.43	0.43	0.43
Aqueous anionic dispersion of a styrene–acrylic copolymer	5.40	5.40	5.40	5.40
Omyadol^®^ 40-JA	29.00	29.00	29.00	29.00
Organomodified polysiloxane-based silicone additive	0.53	0.53	0.53	0.53
Stabilising additive for silicate-based renders	0.33	0.33	0.33	0.33
Propylene glycol	0.10	0.10	0.10	0.10
Non-ionic polyurethane associative thickener used to control the rheology of the silicate render	0.02	0.02	0.02	0.02
Potassium silicate solution	7.60	7.60	7.60	7.60
Sand quartz 0.1–0.5	8.50	8.50	8.50	8.50
Arbocel B400	0.33	0.33	0.33	0.33
Omyadol 0.5–1 mm	9.00	4.5	2.25	0.00
Omyadol 1–1.5 mm	30.00	15.0	7.50	0.00
Waste calcareous opoka rock	0.00	19.50	29.25	39.00

**Table 3:** Composition of the mineral renders, expressed as mass percentages of the total wet formulation.

Constituents	M1 wt.%	M2 wt.%	M3 wt.%	M4 wt.%
Cement CEM I 52.5R white	12.00	12.00	12.00	12.00
Hydrated lime	8.00	8.00	8.00	8.00
Finely ground natural calcium carbonate powder	10.00	10.00	10.00	10.00
Sand quartz	18.63	18.63	18.63	18.63
Omyadol 0.5–1 mm	15.01	11.28	7.52	0.00
Omyadol 1–1.5 mm	34.99	26.235	17.49	0.00
Cellulose ether based on methylcellulose derivatives	0.16	0.16	0.16	0.16
Attapulgite-based inorganic rheology modifier	0.08	0.08	0.08	0.08
Redispersible ethylene–vinyl acetate copolymer powder	1.00	1.00	1.00	1.00
Calcium stearate used as a hydrophobic admixture	0.12	0.12	0.12	0.12
Waste calcareous opoka rock	0.00	12.55	25.1	50.2

**Table 4:** Application properties of fresh render mixtures: resistance to manual texturing and sensitivity to additional water.

Properties of the Fresh Renders		Silicate Renders				Mineral Renders			
		S1	S2	S3	S4	M1	M2	M3	M4
Resistance to manual surface texturing		1	2	2	3	1	2	2	3
Flow value after the addition of water [cm]	+2.5% water	26	24	23	22	24	20	20.5	20
		0.6 *	0.5 *	0.3 *	0.3 *	0.02 *	0.2 *	0.3 *	0.1 *
	+7.5% water	-	29	28	27	-	28	27	27
		-	0.4 *	0.3 *	0.3 *	-	0.2 *	0.2 *	0.3 *

Note: * standard deviation.

**Table 5:** Adhesion to the substrate and impact resistance of the investigated renders.

Parameters	Silicate Plasters				Mineral Plasters			
	S1	S2	S3	S4	M1	M2	M3	M4
Adhesion to the substrate (N/mm^2^)	0.27	0.28	0.29	0.30	0.31	0.26	0.20	0.19
	0.01 *	0.02 *	0.01 *	0.02 *	0.02 *	0.02 *	0.03 *	0.01 *
Impact resistance	No damage or plaster detachment				Plaster detachment and damage			

Note: * standard deviation.

**Table 6:** Initial values of capillary water absorption and adhesion strength, and their ranges of change after accelerated UV exposure and wind-driven rain.

Series	Initial Absorption [kg/(m^2^·24 h)]	Increase in Absorption ΔA [%]	Initial Adhesion Strength [N/mm^2^]	Decrease in Adhesion Strength: Δf_adh_ [%]
S1	1.55	10–20	0.27	8–15
S2	0.68	5–12	0.28	5–10
S3	0.25	3–8	0.29	2–7
S4	0.30	8–15	0.,30	5–10
M1	17.2	8–15	0.31	6–12
M2	17.5	10–18	0.26	8–15
M3	17.9	12–20	0.20	10–20
M4	26.2	18–30	0.19	15–30

**Table 7:** Multi-criteria performance ranking of silicate and mineral renders.

Series	Matrix	WCOR [%]	PI	Application Resistance	Interpretation
S4	Silicate	100	0.796	3	durability-driven optimum
S3	Silicate	75	0.655	2	balanced-performance optimum
S2	Silicate	50	0.480	2	intermediate performance
S1	Silicate	0	0.224	1	Reference
M4	Mineral	100	0.619	3	durability-driven optimum
M2	Mineral	25	0.489	2	balanced option: adhesion/moisture priority
M3	Mineral	50	0.483	2	balanced option: durability priority
M1	Mineral	0	0.429	1	Reference

**Table 8:** Weighting scenarios used in the sensitivity analysis.

Criterion	Equal Weights	Durability-Oriented	Moisture/Adhesion-Oriented
Capillary water absorption	0.143	0.091	0.200
S_d_	0.143	0.091	0.200
Freeze–thaw cycles	0.143	0.182	0.100
Freeze–thaw mass loss	0.143	0.182	0.100
Salt-crystallisation cycles	0.143	0.182	0.100
Salt-crystallisation mass loss	0.143	0.182	0.100
Adhesion	0.143	0.091	0.200

**Table 9:** Sensitivity of the selected optimum to criterion weighting and the practical application constraint AR ≤ 2.

Weighting Scenario	Best Silicate	Best Silicate with AR ≤ 2	Best Mineral	Best Mineral with AR ≤ 2
Equal weights (7 criteria)	S4	S3	M4	M2
Durability-oriented	S4	S3	M4	M3
Moisture/adhesion-oriented	S4	S3	M4	M2

## Data Availability

The datasets generated and/or analysed during the current study are not publicly available but are available from the corresponding author on reasonable request.

## Supplementary Materials

The following supporting information can be downloaded at: https://www.mdpi.com/article/10.3390/ma19183903/s1, Supplementary File S1: Detailed description of the experimental methodology.
