# Supplementary material for: Effect of Waste Calcareous Opoka Rock on the Rheological, Physical, and Durability Properties of Thin-Layer Renders for External Thermal Insulation Composite Systems
Source: Materials (Basel). 2026 Sep 14;19(18):3903. doi: 10.3390/ma19183903 (PMC13608141; doi:10.3390/ma19183903)
Supplement: Supplementary file 1 [file materials-19-03903-s001.zip › File S1-Detailed description of the experimental methodology.pdf]

S1. Methods

S1.1. Testing of fresh mixtures

S1.1.1. Bulk density

Fresh-mortar bulk density was determined in accordance with PN-EN 1015-6 [24]. A clean and dry measuring vessel of known volume V was weighed to determine its mass m_1. The vessel was then filled with fresh mortar and compacted according to the standard procedure. Excess material was removed, the surface was struck off level with the top of the vessel, and the filled vessel was weighed to determine m_2. Fresh-mortar bulk density was calculated using Equation (S1):

$$D = (m_2 - m_1)/V \quad \text{(S1)}$$

where D is the fresh-mortar bulk density (kg/m^3), m_1 is the mass of the empty vessel (kg), m_2 is the mass of the vessel filled with fresh mortar (kg), and V is the vessel volume (m^3). Three determinations were performed for each mortar.

S1.1.2. Consistency

The consistency was determined in accordance with standard PN-EN 1015-3 [25]. The test was carried out using a slump table (Fig. 4). Before each test, the disc and the edges of the mould were wiped with a damp cloth. Once dry, the surfaces were coated with a non-resinous mineral oil of very low viscosity. The mould was placed centrally on the disc of the slump table. The mortar was applied in two layers; each layer was compacted with 10 strikes of a tamper to ensure uniform filling of the mould. Excess mortar was scraped off using a flat scraper. After approximately 15 seconds, the mould was slowly removed. The mortar was distributed across the disc by shaking the flow table 15 times at a constant shaking frequency. The resulting flow diameter was measured in two perpendicular directions, and the mean value was recorded. Three determinations were performed for each formulation, and the results are reported as mean ± standard deviation.

S1.1.3. Resistance during manual surface texturing

Resistance during manual surface texturing was evaluated using an author-developed qualitative comparative procedure. The assessment was not based on a standardised test method and was intended only to compare the application-related behaviour of the investigated fresh render mixtures. Each mixture was applied to the same type of substrate using the same application procedure to obtain a visually uniform layer of comparable thickness. The surface was then textured using the same stainless-steel trowel. All assessments were performed by the same operator under comparable environmental conditions and at the same time after mixing.

The assessment considered resistance to trowel movement, ease of spreading and surface formation, and the perceptibility of aggregate particles beneath the tool. The following three-level ordinal scale was used:

1 – low resistance: the mixture was easily spread and textured, and the trowel moved freely over the surface with negligible resistance. Aggregate particles were not clearly perceptible beneath the tool.

2 – moderate resistance: slight but clearly perceptible resistance occurred during trowelling. The mixture remained readily spreadable and could be textured without excessive force. Aggregate particles were only slightly perceptible beneath the tool.

3 – high resistance: pronounced resistance to trowel movement was observed, and greater force was required to spread and texture the mixture. Aggregate particles were clearly perceptible beneath the tool. The mixture could still be applied and textured, although obtaining a uniform surface required greater effort.

Because the procedure was qualitative and operator-dependent, the resulting scores were treated as ordinal application-related observations. They were not interpreted as direct measurements of rheological properties and were not included as quantitative variables in the calculation of the performance index.

S1.1.4. Mixture sensitivity to excess water

A test of the sensitivity of the mixtures to excessive water addition was carried out to assess changes in their consistency and stability following an increase in the amount of mixing water. Water was added to each mixture in stages, in quantities corresponding to a specific percentage of its mass, and the mixture was then thoroughly mixed until a homogeneous consistency was achieved.

After each stage of water addition, a slump test was carried out in accordance with the procedure used for fresh mortar. The mixture was placed in a mould on the slump table, compacted, the mould removed, and then subjected to 15 impacts. The slump diameter was measured and an assessment was made as to whether the mixture had retained its cohesion, homogeneity and ability to form a proper texture.

A condition in which the mix exhibited excessive spread, loss of stability, segregation of components or the inability to be reliably measured was considered to exceed the acceptable limit. The test allowed a comparison of the susceptibility of individual formulations to a deterioration in workability as a result of adding an excessive amount of water. The resulting flow diameter was measured in two perpendicular directions, and the mean value was recorded. Three determinations were performed for each formulation, and the results are reported as mean ± standard deviation.

S1.2. Testing of the hardened plasters

S1.2.1. Capillary water absorption coefficient

The test was carried out in accordance with the requirements of standard PN-EN 1015-18 [26].

Specimens were dried to constant mass m_0 (kg) and the test face A_s m^2 was immersed to 5–10 mm (Fig. 6). After 24 h, surface water was removed and m_{24} (kg) was recorded. Absorption W_A (kg/m^2 ·24 h) was calculated as:

$$W_A=(m_{24}-m_0)/A_s \quad (S2)$$

Six specimens were tested and results are reported as mean±SD

S1.2.2. Vapour permeability

The diffusion resistance coefficient of the plaster mortar was measured using the 'wet cup' method under isothermal conditions, by placing the test specimen between two different environments with differing relative humidity levels in the surrounding air (PN-EN ISO 12572:2016-10 [27]). The specimens were sealed over test cups containing distilled water, and the assembled cups were placed in a desiccator under isothermal conditions. The cups were weighed at regular intervals to determine the change in mass resulting from water-vapour transmission through the specimens. The water-vapour resistance factor μ was determined in accordance with the calculation procedure specified in the standard. The water-vapour diffusion-equivalent air-layer thickness was calculated as:

$$S_d = \mu d \quad (S3)$$

where S_d is the water-vapour diffusion-equivalent air-layer thickness (m), μ is the dimensionless water-vapour resistance factor, and d is the specimen thickness, equal to 3 mm. Three specimens were tested for each formulation, and the results are reported as mean ± standard deviation.

S1.2.3. Freeze-thaw resistance

The frost resistance test was carried out using the modified direct method in accordance with the procedure described in standard PN-88/B-06250 [28]. This method had to be modified due to the specifications of the thin-layer plasters (it was not possible to produce

blocks measuring 40 × 40 × 160 mm). The plaster was applied to freshly cast concrete blocks. Three samples from each batch were selected for testing. The samples were subjected to cyclic freezing in air at a temperature of –18 ±2 °C for at least 4 hours, followed by thawing in water at a temperature of 18 ±2 °C for 2. Each freeze–thaw cycle constituted one test cycle. The specimens were removed from the chamber every 24 h and visually inspected for cracking and partial or complete detachment of the render layer. If no cracking or detachment was observed, the specimen was returned to the chamber and freeze–thaw cycling was continued.

For this comparative procedure, the failure criterion was defined as the first occurrence of at least one visually detectable crack or any partial or complete detachment of the render layer. The inspection therefore provided a binary assessment: no visible damage or visible damage. The number of completed freeze–thaw cycles at the first inspection during which damage was identified was recorded separately for each specimen. Owing to the 24 h inspection interval, the reported value represents the number of completed cycles at which damage was first detected rather than the exact cycle during which damage initiated.

After completion of the test, specimens that did not exhibit cracking or detachment were removed from the chamber, dried to constant mass, and examined. Their mass loss was calculated relative to the initial dry mass.

The modified procedure was intended to compare the investigated render formulations under identical laboratory conditions. The render–concrete specimens did not reproduce a complete ETICS assembly comprising thermal insulation, an adhesive or reinforced base coat, reinforcement mesh, primer, and finishing render. In particular, the rigid concrete substrate did not reproduce the thermal deformation, moisture transport, or mechanical compliance of an insulation–base-coat assembly. Therefore, the results characterise the comparative freeze–thaw behaviour of render–concrete specimens and should not be interpreted as a direct assessment of the freeze–thaw resistance or service life of a complete ETICS.

S1.2.4. Resistance to the crystallisation pressure of salts

Salt-crystallisation resistance was determined using a modified procedure based on PN-EN 12370:2001 [29]. Because free-standing thin-layer render specimens could not be prepared, the renders were applied to concrete blocks. Six specimens of each formulation were tested. After drying, the specimens were immersed in a 14% sodium sulphate decahydrate solution for 2 h and then gradually heated to 105°C over 10 h. This sequence constituted one cycle. Although the standard specifies 15 cycles, the procedure was continued until the first visible cracking, scaling, or detachment of the render. The number of cycles to first visible damage was recorded. The specimens were then immersed in water for 24 h, rinsed, dried to constant mass, and weighed. Mass loss was calculated relative to the initial dry mass and reported as mean ± standard deviation.

S1.2.5. Adhesion of plasters to the substrate

The test of the adhesion of plasters to the substrate was carried out in accordance with the recommendations of standard PN-EN 1015-12 [30]. Adhesion was defined as the maximum tensile stress induced by a peel load applied perpendicular to the surface of the mortar. The peel load was applied using a peel plate bonded to the exposed surface of the mortar under test. Five measurements were performed for each formulation, and the results are reported as mean ± standard deviation. A concrete paving block was used as the substrate. The test procedure is illustrated in Figure 7.

S1.2.6. Impact resistance of plaster

Impact behaviour (Fig. 8) was evaluated using a modified falling-ball procedure. Before impact, a cross-shaped cut was made in the render surface using an angle grinder to introduce an initial discontinuity. The experimental procedure was based on the general

principle of the hard-body impact test used for rendered external thermal insulation composite systems (ETICS), as described in PN-EN 13497 + A1:2021-06 [31], Thermal insulating products for buildings - Determination of impact resistance of external thermal insulation composite systems (ETICS)] and in the EAD/ETAG assessment procedures for ETICS. A steel ball with a mass of 0.405 kg was then dropped vertically onto the intersection of the cuts from a height of 754 mm, corresponding to an impact energy of approximately 3 J:

$$E = mgh = 0.405 \times 9.81 \times 0.754 = 3.0 \text{ J} \quad \text{(S4)}$$

where E is the impact energy, m is the ball mass, g is gravitational acceleration, and h is the drop height.

Therefore, the paper does not present the procedure as a standard test and does not assign the investigated materials to standard ETICS impact-resistance categories. Before impact, a cross-shaped cut was introduced into the surface of each render using an angle grinder (Fig. 8). The steel ball was then dropped onto the intersection of the cuts. The cut acted as an artificially introduced initial discontinuity and enabled the behaviour of the render in the presence of existing damage to be evaluated. In contrast to a standard impact applied to an intact surface, the procedure was primarily intended to examine the subsequent development of damage rather than its initial formation.

After impact, the specimens were visually examined for crack extension from the ends of the cuts, local spalling, and partial or complete detachment of the render layer from the substrate.

S1.2.7. UV aging test

Ultraviolet radiation is characterized by sufficiently high photon energy to initiate the degradation of selected chemical bonds present in organic coating components. Following absorption of UV radiation, polymeric materials may undergo bond scission, photolytic degradation and photo-oxidation, leading to progressive changes in their chemical structure and functional performance. Prolonged UV exposure may also weaken the interfacial bond between the coating and the substrate, thereby reducing adhesion and diminishing the hydrophobic properties of the protective layer.

The resistance of the coatings to UV-induced degradation was evaluated using a TestAn Xentest 2200 xenon-arc weathering chamber (TestAn, Gdańsk, Poland). The exposure conditions were selected to reproduce, in an accelerated manner, the effect of approximately one year of outdoor solar radiation under climatic conditions representative of Poland. The adopted test parameters were as follows: total exposure time of 720 h, chamber temperature of 63 °C and irradiance of 0.51 W/m². The results were expressed as the change in capillary water absorption after ageing relative to the initial value and as the reduction in adhesion strength. A positive value of the index indicates an increased susceptibility of the render to liquid water transport and, consequently, a deterioration in its resistance to moisture exposure. The change in capillary water absorption was calculated according to the following equation:

$$\Delta A = [(A_{after} - A_{before}) / A_{before}] \times 100\% \quad \text{(S5)}$$

where A_{before} is is the capillary water absorption measured before ageing, kg/(m²·24 h); A_{after} is is the capillary water absorption measured after ageing, kg/(m²·24 h).

The reduction in adhesion strength was determined as the relative decrease in the bond strength Δf_{adh} between the render and the substrate:

$$\Delta f_{adh} = [(f_{before} - f_{after}) / f_{before}] \times 100\%. \quad \text{(S6)}$$

where f_{before} is the adhesion strength measured before ageing, N/mm²; f_{after} is the adhesion strength measured after ageing, N/mm².

The greater the value of Δf_{adh}, the more pronounced the degradation of the render–substrate interface resulting from exposure to UV radiation, cyclic wetting and drying, and moisture fluctuations.

S1.2.8. Microstructure – scanning microscopy

The observations were carried out using a Quanta 250 FEG microscope (FEI, Hillsboro, OR, USA) equipped with an EDAX energy-dispersive X-ray spectroscopy (EDS) system for chemical composition analysis. The SEM samples, in the form of fragments, were mounted on a carbon holder using carbon adhesive. The specimens were then sputter-coated with a layer of carbon approximately 50 nm thick in a sputtering chamber to achieve surface conductivity. Two magnifications were used: 500x and 5000x.
